# GABA_B_ Receptor Agonist R-Baclofen Reverses Altered Auditory Reactivity and Filtering in the *Cntnap2* Knock-Out Rat

**DOI:** 10.3389/fnint.2021.710593

**Published:** 2021-08-20

**Authors:** Dorit Möhrle, Wenxuan Wang, Shawn N. Whitehead, Susanne Schmid

**Affiliations:** Department of Anatomy and Cell Biology, Schulich School of Medicine & Dentistry, University of Western Ontario, London, ON, Canada

**Keywords:** autism spectrum disorders, sensory processing, startle, GABA, R-Baclofen, *CNTNAP2*, animal model

## Abstract

Altered sensory information processing, and auditory processing, in particular, is a common impairment in individuals with autism spectrum disorder (ASD). One prominent hypothesis for the etiology of ASD is an imbalance between neuronal excitation and inhibition. The selective GABA_B_ receptor agonist R-Baclofen has been shown previously to improve social deficits and repetitive behaviors in several mouse models for neurodevelopmental disorders including ASD, and its formulation Arbaclofen has been shown to ameliorate social avoidance symptoms in some individuals with ASD. The present study investigated whether R-Baclofen can remediate ASD-related altered sensory processing reliant on excitation/inhibition imbalance in the auditory brainstem. To assess a possible excitation/inhibition imbalance in the startle-mediating brainstem underlying ASD-like auditory-evoked behaviors, we detected and quantified brain amino acid levels in the nucleus reticularis pontis caudalis (PnC) of rats with a homozygous loss-of-function mutation in the ASD-linked gene *Contactin-associated protein-like 2* (*Cntnap2*) and their wildtype (WT) littermates using Matrix-Assisted Laser Desorption Ionization Mass Spectrometry (MALDI MS). Abnormal behavioral read-outs of brainstem auditory signaling in *Cntnap2* KO rats were accompanied by increased levels of GABA, glutamate, and glutamine in the PnC. We then compared the effect of R-Baclofen on behavioral read-outs of brainstem auditory signaling in *Cntnap2* KO and WT rats. Auditory reactivity, sensory filtering, and sensorimotor gating were tested in form of acoustic startle response input-output functions, short-term habituation, and prepulse inhibition before and after acute administration of R-Baclofen (0.75, 1.5, and 3 mg/kg). Systemic R-Baclofen treatment improved disruptions in sensory filtering in *Cntnap2* KO rats and suppressed exaggerated auditory startle responses, in particular to moderately loud sounds. Lower ASR thresholds in *Cntnap2* KO rats were increased in a dose-dependent fashion, with the two higher doses bringing thresholds close to controls, whereas shorter ASR peak latencies at the threshold were further exacerbated. Impaired prepulse inhibition increased across various acoustic prepulse conditions after administration of R-Baclofen in *Cntnap2* KO rats, whereas R-Baclofen did not affect prepulse inhibition in WT rats. Our findings suggest that GABA_B_ receptor agonists may be useful for pharmacologically targeting multiple aspects of sensory processing disruptions involving neuronal excitation/inhibition imbalances in ASD.

## Introduction

Autism spectrum disorders (ASD) are neurodevelopmental disorders characterized by behavioral deficits in social interaction and unusual social communication as well as stereotyped, repetitive behaviors with restricted interests including sensory issues (DSM-5, 2013). Sensory abnormalities are present in over 90% of children with autism and can lead to great distress in everyday life settings (O’Neill and Jones, 1997; Leekam et al., 2007). Impairments in pre-attentive filtering of inundating sensory information, for example in noisy environments (Ornitz et al., 1993; Braff et al., 2001; Perry et al., 2007; Stevenson et al., 2017), are often accompanied by increased loudness perception (Khalfa et al., 2004; Danesh et al., 2015) and exaggerated reflexive responses to sudden sounds (Chamberlain et al., 2013; Kohl et al., 2014; Takahashi et al., 2016). The neural basis underlying ASD-related differences in sensory and other symptomatic behaviors has been hypothesized to be an imbalance in excitation and inhibition (E/I; Rubenstein and Merzenich, 2003). Indeed, alterations in biomarkers for GABA and glutamate (Glu) abundance and neurotransmission have been described in humans with ASD as well as in a multitude of rodent models with targeted mutations in risk genes for ASD (e.g., Yip et al., 2008; Blatt and Fatemi, 2011; Harada et al., 2011; Coghlan et al., 2012; Sgadò et al., 2013; Gaetz et al., 2014; Bridi et al., 2017; Horder et al., 2018b). Treatment options for ASD are currently limited, although pharmacological agents that modulate E/I balance showed promising preliminary results in clinical trials (for review, see Oberman, 2012; Port et al., 2019). As such, the selective GABA_B_ receptor agonist Arbaclofen or its formulation R-Baclofen has been shown to ameliorate social avoidance symptoms in some individuals with ASD or the related genetic disorder Fragile X Syndrome (Berry-Kravis et al., 2012, 2017; Erickson et al., 2014; Veenstra-VanderWeele et al., 2017) and to improve social behavior deficits and repetitive behaviors in several corresponding genetic mouse models (Henderson et al., 2012; Silverman et al., 2015; Sinclair et al., 2017a; Stoppel et al., 2018). However, to the best of our knowledge, to date, no preclinical or clinical study has thoroughly investigated the potential of R-Baclofen for the treatment of behavioral outcomes of sensory abnormalities associated with ASD.

Homozygous loss-of-function mutations in the Contactin Associated Protein-like 2 (*Cntnap2*) gene have been identified as one of the rare single gene causes for ASD (Strauss et al., 2006; Poot, 2017). The protein encoded by *Cntnap2*, the neurexin CASPR2, shows enriched expression in sensory pathways of the brain (Gordon et al., 2016). CASPR2 is involved in neurotransmitter release and excitability through its clustering of voltage-gated potassium channels located at the juxtaparanodes of myelinated axons, at axonal segments, and synaptic terminals (Poliak et al., 2003; Scott et al., 2017). Rats and mice with knockout of the *Cntnap2* gene display alterations in sensory processing, both on the neuronal and behavioral level (Peñagarikano et al., 2011; Truong et al., 2015; Scott et al., 2017, 2020; Townsend and Smith, 2017; Dawes et al., 2018). In particular, alterations in brainstem auditory processing and auditory reactivity (Scott et al., 2018, 2020) reflect those reported in individuals with ASD (for review, see Sinclair et al., 2017b).

**Figure 1:**
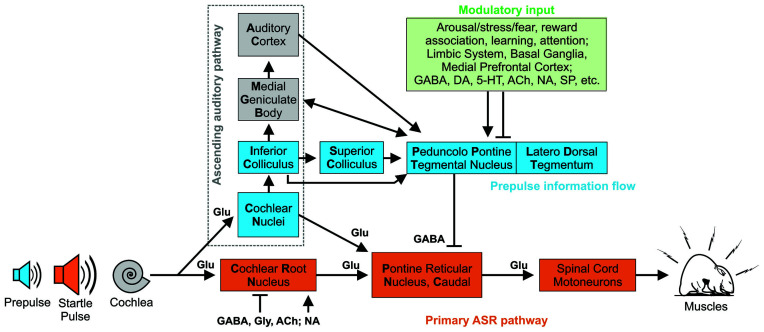
Hypothetical simplified primary and modulatory neural circuitry underlying acoustic startle response and prepulse inhibition with presumed or confirmed neurotransmitters. In the primary ASR pathway (orange), the startle pulse sound information is transmitted *via* the auditory nerve to cochlear root neurons (CRN) in rodents which give short-latency input to the PnC (Pontine Reticular Nucleus, Caudal). These neurons project directly to cranial and spinal cord motoneurons to produce the motor response. Multiple pathways have been identified for sensorimotor gating modulating ASR. The acoustic prepulse is processed through the ascending auditory pathway (gray dotted frame) *via* the cochlear nucleus (CN) and the inferior colliculus. From there the prepulse information is transmitted directly or indirectly to the pedunculopontine tegmental nucleus (PPT) and neighboring region laterodorsal tegmental nucleus. Projections from the PPT to the PnC mediate PPI as a form of feed-forward inhibition. The PPT receives modulatory input (green pathway) from a variety of brain structures that can influence PPI e.g., linked with fear and arousal. Abbreviations: ACh, Acetyl choline; GABA, gamma aminobutyric acid; Glu, Glutamate; Gly, Glycine; DA, Dopamine; 5-HT, Serotonine; NA, Noradrenaline, SP, Substance P. Based on data and information from Lauer et al. (2017), Fulcher et al. (2020), and Gómez-Nieto et al. (2020).

In the present study, we investigated if selective activation of GABA_B_ receptors can remediate ASD-related altered sensory processing reliant on auditory brainstem function. We compared deficits in behavioral measures of auditory brainstem function from adult female and male *Cntnap2* knockout (KO) and wildtype (WT) controls after acute administration of vehicle (saline) or three different doses of R-Baclofen (0.75, 1.5, 3 mg/kg). Reflexive responses to startle-eliciting sounds were used to determine the efficacy of R-Baclofen to normalize acoustic reactivity, sensory filtering (i.e., short-term habituation), and sensorimotor gating (i.e., prepulse inhibition, PPI) in *Cntnap2* KO rats. The implicit (reflexive) reactivity to acoustic stimuli is mediated by a short primary pathway in the lower brainstem that activates spinal motor neurons to produce the startle response (Figure 1). An important element of the startle pathway is the nucleus reticularis pontis caudalis (PnC), the sensorimotor interface where cochlear root neurons (CRN) synapse on premotor neurons. Importantly, the transition of sensory input into the motor output can be directly influenced in the PnC by excitatory or inhibitory afferents (for review, see Koch, 1999; Simons-Weidenmaier et al., 2006). To further determine how R-Baclofen affects the transduction of sensory input into motor output within the brainstem startle circuit, we determined the threshold, effective stimulus (ES50), saturation, and slope of the startle input-output (ASR I-O) functions, as well as startle peak latencies. Finally, we quantified GABA, Glu, and glutamine (Gln) levels in the startle mediating PnC from *Cntnap2* KO and WT rats using Matrix-Assisted Laser Desorption Ionization Mass Spectrometry (MALDI MS) to determine if E/I imbalance underlies ASD-like deficits in brainstem auditory processing and behavior.

Overall, the present study provides preclinical evidence that acute, systemic R-Baclofen treatment reverses many disruptions in brainstem-mediated auditory processing and behavior associated with mutations in the autism-linked gene *Cntnap2*. These findings support further investigations of GABA_B_ receptor agonists as promising pharmacological targets for the rescue of sensory processing deficits seen in neurodevelopmental disorders including ASD.

## Materials and Methods

### Animals

Male (M) and female (F) adult Sprague–Dawley wildtype (*Cntnap2* WT) and homozygous knockout (*Cntnap2* KO) rats were used in this study. Heterozygous breeders were obtained from Horizon Discovery (Boyertown, PA, USA), and all experimental animals were obtained from heterozygous crossings. Rats were housed in a temperature-controlled room on a 12 h light/dark cycle with *ad libitum* food and water. Behavioral testing was performed during the light phase of the cycle (lights on at 07:00 h). All procedures were approved by the University of Western Ontario Animal Care Committee and were in accordance with the guidelines established by the Canadian Council on Animal Care.

### Acoustic Startle Responses (ASRs)

**Figure 2:**
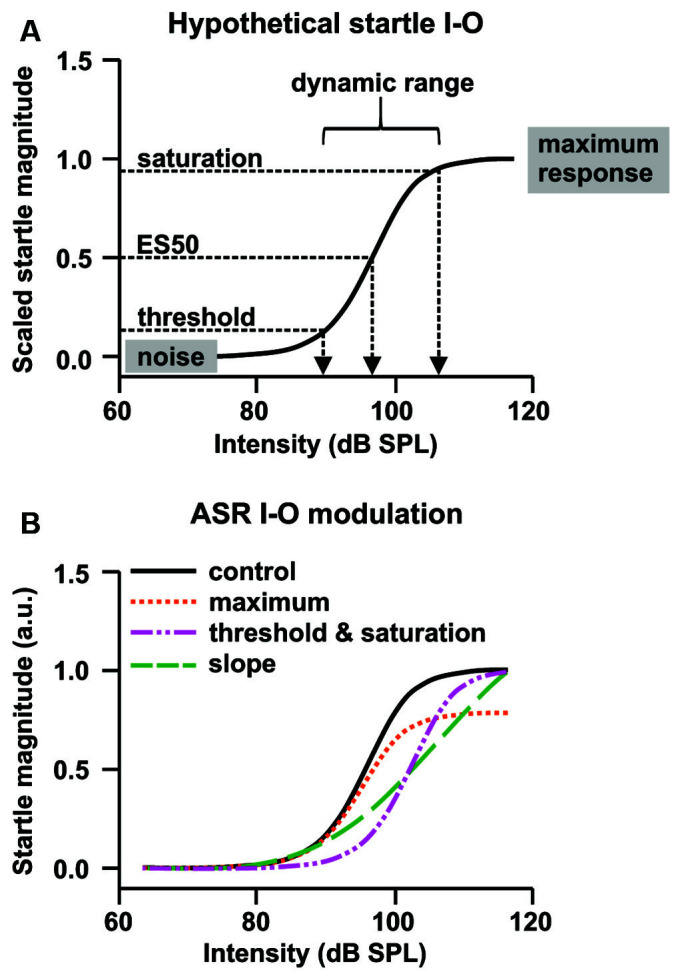
Hypothetical plasticity of ASR input-output (I-O) functions. **(A)** The sigmoid function describing the relationship between startle stimulus (input) and startle response magnitude (output). The ASR threshold is a putative measure for ASR excitability, the efficient stimulus (ES) for the sound pulse potency, and the ASR maximum for motor capacity. Within the dynamic range of the I-O function, a small stimulus change can produce a large response change. The slope of the dynamic range can be used as metric for the reflex efficiency.** (B)** The control ASR I-O function (black solid line) could be altered through an increase or decrease in the maximum response to the loudest startle pulse (orange), a left- or right-shift of the curve, thereby increasing or decreasing ASR threshold, ES50, and saturation (pink), a steepening or flattening of the slope of the dynamic range of the function (green), or a combination of these effects (based on data and information from Hince and Martin-Iverson, 2005; Martin-Iverson and Stevenson, 2005). Normalization of the ASR I-O function to the individual startle magnitude at the loudest startle pulse allows analysis of threshold and slope without confounding effects of altered maximum response.

To investigate the effects of R-Baclofen on ASRs to startle-eliciting sounds, rats of the two genotypes (WT: 6 F, 5 M; KO: 6 F, 5 M) were tested after injection of 0.75, 1.5, and 3 mg/kg *i.p*. R-Baclofen at 8- to 11-months of age. The assessment of acoustic reactivity, sensory filtering, and sensorimotor gating was conducted in sound-attenuating startle boxes (LE116; Panlab) using the StartFear system (Panlab) and STARTLE software module (PACKWIN-CSST, PACKWIN version 2.0; Panlab) as described (Scott et al., 2018, 2020). In brief, using a pressure-sensitive platform, the rat’s acoustic reactivity was measured as the magnitude of its startle response to acoustic stimuli (ASR I-O function) at varying intensities [pulse: 20 ms, 65–115 dB SPL in 5 dB steps, 10 stimuli of each in randomized order, inter-trial interval (ITI): 15, 17.5, or 20 s during a continuous background noise 60 dB SPL white noise]. To determine the startle threshold, effective stimulus ES50, and saturation (Figure 2) of each animal, we first scaled the ASR I-O function of a given animal and treatment between 0 and 1, then fit the scaled function with a GraphPad Prism 8.4.3 in-built model (Model: Standard curves to interpolate—Sigmoidal, 4PL, X is concentration; Method: Prism’s default parameters; Compare: “Do the best-fit values of selected unshared parameters differ between data sets?,” Comparison method: Extra sum-of-squares F test, Parameters: Bottom, Top, ES50, HillSlope; Constrain: constrain bottom to 0, top to 1; Initial values: choose automatically) with the following equation:

$$Y=Botom+(X^{HillSlope})*\frac{Top-Bottom}{X^{HillSlope}+ES50^{HillSlope}}$$

*Y* is the ASR magnitude

*Bottom* is the lower plateau of the startle pulse intensity (dB SPL) on the Y axis

*Top* is the upper plateau of the startle pulse intensity (dB SPL) on the Y axis

*HillSlope* is the steepness of the slope

*ES50* is the startle pulse intensity that gives an ASR magnitude halfway between Bottom and Top.

We then solved the above equation for *X* and calculated the ASR threshold and saturation for each animal from their individual curve fit parameters using MATLAB R2019a:

$$X=\sqrt[{HillSlope}]{\frac{(Y-Bottom)*ES50^{HillSlope}}{Top-Y}}$$

The ASR threshold *X* was defined at *Y* equal to 25% and the ASR saturation at *Y* equal to 90% of the span between the Top and Bottom plateau. We chose these values because the estimated ASR threshold and saturation after saline injection matched the between startle pulse intensity comparisons within genotype. ASR peak latencies were extracted within the I-O dynamic range (i.e., we rounded up threshold and rounded down saturation estimates to extract latencies of responses to actually measured sound pulse intensities).

Sensorimotor gating (expressed as the percentage of prepulse inhibition, %PPI) was determined by the extent that the rat’s startle response to the 105 dB SPL pulse was attenuated when a brief prepulse was presented 30 or 100 ms prior to the startle stimulus (prepulse: 10 ms, 65, 75, or 85 dB SPL). Because startle reactivity can affect sensorimotor gating (Csomor et al., 2008), differences in baseline startle magnitude were calculated using the startle-only trials during PPI blocks and analyzed. The control startle stimulus (105 dB SPL) without prepulse and each combination of prepulse lead time and intensity was presented 10 times. The relative percentage of PPI was calculated using the maximum startle amplitudes as follows:

$$\%PPI=\left(1-\frac{prepulse\;pulse}{pulse\;alone}\right)*100\%$$

The latency of the startle response was also measured in trials with/without the prepulse, as an increase in startle latency in PPI trials is typical for sensorimotor gating (Ison et al., 1973; Hoffman and Ison, 1980). Relative changes in latency were calculated as the time to reach the maximum startle magnitude on startle pulse-alone trials subtracted from that during prepulse trials, i.e., positive values represented a latency increase (Lyall et al., 2009).

To determine the impact of R-Baclofen on sensory filtering, *Cntnap2* WT and KO rats were repeatedly presented a startle-eliciting stimulus (20 ms white noise at 105 dB SPL; 5 ms rise/fall time, ITI: 15, 17.5, or 20 s during a continuous background noise 60 dB SPL white noise) and the degree that their startle response habituated was compared across the genotypes and treatments. Habituation was assessed from the first eight trials with the startle magnitudes normalized to the magnitude at the first trial. A habituation score was calculated for each animal using the following formula (Scott et al., 2018):

$$Habituation\;score=\frac{average\;\max\;startle\;magnitude\;trials\;7\;and\;8}{\max\;startle\;magnitude\;trial\;1}$$

A sensitization score was calculated for each animal using the following formula (Meincke et al., 2004):

$$Sensitization\;score=\frac{average\;\max\;startle\;magnitude\;trials\;2\;and\;4}{\max\;startle\;magnitude\;trial\;1}$$

Before the behavioral procedures associated with the ASR (i.e., acoustic reactivity, sensory filtering, and sensorimotor gating), animals were handled and acclimated to the startle boxes over three 10 min sessions. During these acclimation sessions, only background noise (60 dB SPL, white noise) was presented to the animals.

### Drug Application

R-Baclofen (provided by Simons Foundation Autism Research Initiative, SFARI through Clinical Research Assoc., LLC) was dissolved in 0.9% saline freshly on each experimental day and administered intraperitoneally (i.p., injection volume: 1 ml/kg) 1 h before the start of the test session in doses of 0.75, 1.5, and 3 mg/kg. Doses and injection time before testing were chosen in accordance with the literature (Henderson et al., 2012; Silverman et al., 2015; Lorrai et al., 2016). The vehicle condition was represented by the administration of an equal volume of saline. In the first experimental block (Supplementary Figure 1), each treatment was administered on two consecutive days in the following order: saline (Day 1&2), R-Baclofen at 0.75 mg/kg (Day 3&4), 1.5 mg/kg (Day 5&6), and 3 mg/kg (Day 7&8). We chose to inject R-Baclofen in increasing doses to avoid the need for week-long washout times between treatments (Henderson et al., 2012) and to keep behavioral testing as concise as possible. On the first day of each dose, injections of saline or R-Baclofen were followed by behavioral tests for sensory filtering (habituation) and acoustic reactivity (ASR I-O), and on the second day by the behavioral test for sensorimotor gating (PPI). After the first experimental block and a 1-week washout period, we repeated the same sequence of behavioral procedures over 8 days with saline injection. This second experimental block was used to control for effects of repeated testing. Over 2 days preceding either experimental block, rats were habituated to handling, behavioral testing, and intraperitoneal injections; specifically, rats received one injection of 1 ml/kg saline 1 h prior to testing on both days (not shown in Supplementary Figure 1). No statistical differences were found for either genotype between saline treatments across the two experimental blocks for habituation and acoustic reactivity and data were pooled across days for the most accurate genotype comparisons after saline. For PPI of ASR, genotype comparisons after saline were made based on Day 2 (Supplementary Figure 1) because there was a significant difference between the PPI of the two experimental blocks. Repeated testing within the second experimental block did not alter the PPI in *Cntnap2* WT and KO rats (Supplementary Figure 2) and we assumed that effects of repeated testing within the first experimental block were also negligible. For most consistent comparisons of R-Baclofen treatment effects within or between genotypes, data were compared within the first treatment block.

### MALDI

In order to analyze if altered brain amino acid abundances underlie the *Cntnap2*-linked changes in auditory-evoked behaviors, 8 *Cntnap2* WT (4 F, 4 M) and 8 *Cntnap2* KO (4 F, 4 M) rats were deeply anesthetized with carbon dioxide and decapitated at 4- to 5-months-old. Brains were extracted and fresh frozen, and stored at −80°C, until cryosectioned at 10μm (Thermo-Fisher Scientific CryoStar NX50), and mounted on Indium tin oxide (ITO)-coated glass slides (Hudson Surface Technology Inc., Old Tappan, NJ, USA). Zinc oxide (Sigma-Aldrich, St. Louis, MO, USA) was selected as the MALDI matrix and prepared to 1 mg/ml in 50% ACN and 0.1% TFA (Fisher Scientific, Waltham, MA, USA) and applied onto the slides with TM-Sprayer^TM^ (HTX Technologies, LLC, Chapel Hill, NC, USA). Afterward, α-cyano-4-hydroxycinnamic acid standards were spotted onto the slides for internal mass calibration. ZnO matrix deposition using the TM Sprayer and MS data analysis of neurotransmitters were performed as previously described (Chen et al., 2021). MALDI-MS sample analyses were performed on a Sciex TOF/TOF 5800 MALDI mass spectrometer (Sciex, Framingham, MA, USA). Images were acquired in the positive ion reflectron mode at a mass range of 50–300 m/z using the TOF-TOF Series Explorer and Data Explorer were used for data acquisition and processing, respectively (Sciex). MS images were acquired at 70 μm raster with 50 shots/spectrum, and the laser energy was optimized based on the signal intensity, peak resolution and signal-to-noise ratio. MALDI MS images were visualized and analyzed through an experimentally blinded observer using Tissue View^TM^ Software IDL^VM^ (Sciex). PnC and superior olivary complex (SOC) region of interest in the brainstem were manually selected to generate the average mass spectra. Mass peaks corresponding to neurotransmitters and metabolites ([GABA+K]^+^: 142m/z, [Glu+K]^+^: 186m/z, [Gln+K]^+^: 185m/z, [Choline+K]^+^: 143m/z, [Norepinephrine+K]^+^: 208m/z) were acquired from each mass spectra (Chen et al., 2021). Comparative analysis was performed based on the area under the curve (AUC) ratio (ratio between the peak of interest AUC to total AUC from 100 to 250m/z; Caughlin et al., 2017). Intensity values corresponding to the mass peaks were compared between respective WT-KO pairs on the slides. One female WT-KO pair had to be excluded due to significant tearing in the tissue that left the PnC region of interest unusable, resulting in three pairs of female and four pairs of male *Cntnap2* WT-KO rats. No significant differences between data from females and males were observed and data were pooled.

### Statistical Analysis

Unless otherwise stated, data that followed normal distribution are presented as group mean with standard deviation (SD), and not normally distributed data as group median with interquartile range (IQR). Depending on the experimental design and distribution of the data, differences of the means were compared for statistical significance either by student’s *t-*test, paired *t*-test, Welch *t*-test, one sample *t*-test, one sample Wilcoxon test, Mann–Whitney test, 2-way ANOVA, repeated measures (RM) ANOVA, Mixed-effect analysis, or Friedmann tests using GraphPad Prism 8.4.3 (La Jolla, USA). For 2-way ANOVA comparisons we did not assume sphericity because R-Baclofen was administered in consecutive, increasing doses (Supplementary Figure 1) and we used Greenhouse-Geisser correction where applicable. Two-way ANOVA, RM ANOVA, Mixed-effect analysis, or Friedmann tests were followed by multiple comparison tests with correction for type 1 error after Sidak’s, Dunnett’s, or Dunns’s multiple comparisons test. The relative amount of prepulse inhibition was additionally analyzed by random permutation tests in consideration of small sample sizes to estimate the population mean from samples (resampling by bootstrapping, property mean, 10,000 random samples without replacement). Statistical significance level was *α* = 0.05, and resulting *p* values are reported in the legends using: (*)*p* < 0.1, *p ≤ 0.05; **p ≤ 0.01; ***p ≤ 0.001; n.s., not significant.

## Results

### *Cntnap2* KO Rats Display Deficient Short-Term Habituation, Exaggerated Sensitization, and Increased Acoustic Reactivity

In order to investigate whether selective activation of GABA_B_ receptors can remediate ASD-related altered sensory processing reliant on auditory brainstem function, we analyzed auditory reactivity, filtering, and sensorimotor gating in adult *Cntnap2* KO rats (*n =* 6 F, *n* = 5 M) in comparison to WT littermates (*n =* 6 F, *n* = 5 M) after acute administration of R-Baclofen (0.75, 1.5, and 3 mg/kg) or vehicle (saline).

**Figure 3:**
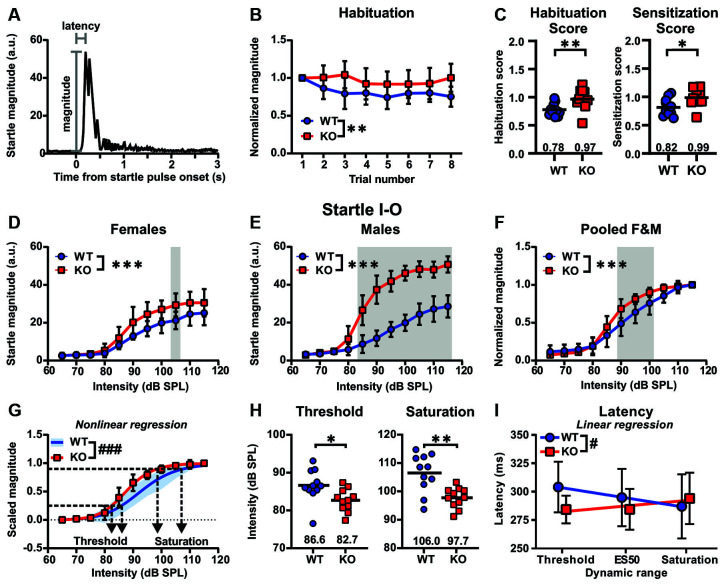
Impaired short-term habituation and increased acoustic reactivity in *Cntnap2* KO compared with wildtype (WT) rats.** (A)** Schematic raw acoustic startle trace to a 115 dB SPL startle pulse. ASR peak magnitude and latency is determined within the recording window of 500 ms from startle pulse onset.** (B,C)** Sensory filtering as measured by short-term habituation is perturbed in *Cntnap2* KO rats. **(B)** Mean ± standard deviation (SD) startle response magnitudes across eight subsequent trials normalized to the first trial in *Cntnap2* WT and KO rats. Values <1 indicate habituation of the startle response. *Cntnap2* KO rats showed less declined startle responses than WT rats, indicating perturbed habituation, in particular at trial number eight (*Cntnap2* WT: *n* = 11, *Cntnap2* KO: *n* = 11, two-way repeated measures (RM) ANOVA, trial × genotype *p* = 0.0225, *F*_(7,140)_ = 2.425, trial *p* = 0.0012, *F*_(4.714, 94.28)_ = 4.528, genotype *p* = 0.0053, *F*_(1,20)_ = 9.792, Sidak’s multiple comparisons test, *p* = 0.0151). **(C) Left**: Individual habituation scores calculated from the average of the last two trials divided by that of the first trial (WT: blue circles, KO: red squares, mean: horizontal black line). Values <1 indicate habituation of the startle response. *Cntnap2* KO rats had significantly greater habituation scores compared with WT rats (two-sided student’s *t*-test *p* = 0.0082).** Right**: Individual sensitization scores calculated from the average of trials 2–4 divided by that of the first trial (WT: blue circles, KO: red squares, mean: horizontal black line). *Cntnap2* KO rats showed greater sensitization scores than WT rats (two-sided student’s *t*-test *p* = 0.0150). **(D–F)** Mean ± SD startle responses in female (F) **(D)**, male (M) **(E)**, and pooled male and female **(C)** *Cntnap2* WT (blue circles) and KO (red squares) rats to startle pulses with sound intensities from 65 to 115 dB SPL in 5 dB steps. **(D)** Acoustic startle magnitudes were significantly increased in female *Cntnap2* KO (WT: *n* = 6, KO: *n* = 6, two-way ANOVA, intensity × genotype *p* = 0.1598, *F*_(10,110)_ = 1.471, intensity *p* < 0.0001, *F*_(10,110)_ = 61.70, genotype* p* < 0.0001, *F*_(1,110)_ = 28.83) and **(E)** male *Cntnap2* KO rats (WT: *n* = 5, KO: *n* = 5, two-way ANOVA, intensity × genotype *p* < 0.0001, *F*_(10,88)_ = 14.97, intensity *p* < 0.0001, *F*_(10,88)_ = 100.1, genotype* p* < 0.0001, *F*_(1,88)_ = 301.7) compared with WT littermates when collapsing over intensities, indicated by a leftward shift of the I-O ASR function. In particular, startle magnitudes were elevated in female *Cntnap2* KO at 105 dB SPL (Sidak’s multiple comparisons test, *p* = 0.0303) and in male *Cntnap2* KO rats at 85–115 dB SPL (Sidak’s multiple comparisons test, all *p* < 0.0001). **(F)** Normalized ASR I-O functions pooled for male and female *Cntnap2* WT and KO rats were significantly different (*Cntnap2* WT: *n* = 11, *Cntnap2* KO: *n* = 11, two-way ANOVA intensity × genotype *p* < 0.0001, *F*_(10,220)_ = 4.750, intensity *p* < 0.0001, *F*_(10,220)_ = 313.5, genotype* p* < 0.0001, *F*_(1,220)_ = 20.63). Normalized startle magnitudes in *Cntnap2* KO rats were particularly increased in comparison to *Cntnap2* WT rats at 90–100 dB SPL (Sidak’s multiple comparisons test, 90 dB SPL: *p* < 0.0001, 95 dB SPL: *p* = 0.0005, 100 dB SPL: *p* = 0.0069). **(G)** Sigmoidal curves (lines) fitted to the startle magnitudes scaled between 0 and 1 were significantly different in *Cntnap2* WT (SD, blue area) and *Cntnap2* KO rats (mean ± SD, red squares and error bars; *p* < 0.0001, curve fit values see Table 1). Dotted horizontal line at 0.25 determined as ASR threshold and at 0.9 as ASR saturation. **(H)** Individual ASR thresholds (**Left**) and saturation (**Right**) extracted from individual sigmoidal curve fits were significantly lower in *Cntnap2* KO rats (blue squares, horizontal black lines: mean) compared with WT controls (red circles, horizontal black lines: mean; two-sided student’s *t*-test, threshold: *p* = 0.0210, saturation: *p* = 0.0012). **(I)** Linear regression of ASR peak latencies across the dynamic range of *Cntnap2* WT (mean ± SD, blue circles and error bars, *Y* = −8.537 * X + 312.4, *r^2^* = 0.9979, blue line) and KO rats (mean ± SD, red squares and error bars, KO: *Y* = 4.910 * X + 277.8, *r^2^* = 0.7576, red line). The slopes of the regression lines were significantly different (*p* = 0.0408). ES50, acoustic startle pulse intensity that gives a startle magnitude at 50%. *p < 0.05; **p < 0.01; ***p < 0.001; ^#^*p* < 0.05 (comparison of regression lines); ^###^*p* < 0.001 (comparison of regression lines).

To most accurately assess genotype-related differences between *Cntnap2* WT and KO rats in sensory filtering and acoustic reactivity (Figure 3), we first averaged the respective data from the five experimental days where short-term habituation and ASR I-O trials were measured 1 h after saline injection (Day # 1, 16, 18, 20, 22, see timeline in Supplementary Figure 1). To assess sensory filtering, short-term habituation of the startle response was measured across the first eight startle trials of the test day. Short-term habituation across the first eight startle trials of the test day revealed significantly less declined startle responses in *Cntnap2* KO compared with WT rats, in particular at trial number eight (*Cntnap2* WT: *n* = 11, *Cntnap2* KO: *n* = 11, two-way RM ANOVA, trial × genotype *p* = 0.0225, *F*_(7,140)_ = 2.425, trial *p* = 0.0012, *F*_(4.714, 94.28)_ = 4.528, genotype *p* = 0.0053, *F*_(1,20)_ = 9.792, Sidak’s multiple comparisons test, *p* = 0.0151, Figure 3B), indicating that KO rats do not habituate across trials. Habituation scores calculated from trial 7 and 8 in relation to trial 1 were significantly increased in *Cntnap2* KO rats compared with WT rats (Figure 3C Left, *Cntnap2* WT: 0.78 ± 0.11, KO: 0.97 ± 0.18 dB SPL, two-sided student’s *t*-test *p* = 0.0082), confirming perturbed habituation across trials and indicating impaired sensory filtering in *Cntnap2* KO rats. Furthermore, *Cntnap2* KO rats displayed greater sensitization scores calculated from trial 2–4 in relation to trial 1 than *Cntnap2* WT rats (Figure 3C Right, *Cntnap2* WT:0.82 ± 0.15, KO:0.99 ± 0.16, two-sided student’s *t-test* *p* = 0.0150).

To assess acoustic reactivity in *Cntnap2* WT and KO rats, startle response magnitudes to a series of startle pulses of increasing intensity (65–115 dB in 5 dB SPL increments) were measured and analyzed. Deficient short-term habituation and exaggerated sensitization in *Cntnap2* KO rats described above were accompanied by increased ASR magnitudes of the startle I-O growth function in both female (Figure 3D) and, even more so, in male (Figure 3E) *Cntnap2* KO rats compared with respective WT controls (F WT: *n* = 6, F KO: *n* = 6, two-way ANOVA, intensity × genotype *p* = 0.1598, *F*_(10,110)_ = 1.471, intensity *p* < 0.0001, *F*_(10,110)_ = 61.70, genotype* p* < 0.0001, *F*_(1,110)_ = 28.83; M WT: *n* = 5, M KO: *n* = 5, two-way ANOVA, intensity × genotype *p* < 0.0001, *F*_(10,88)_ = 14.97, intensity *p* < 0.0001, *F*_(10,88)_ = 100.1, genotype* p* < 0.0001, *F*_(1,88)_ = 301.7). ASR magnitudes were particularly increased at 105 dB SPL, and at 85–115 dB SPL startle pulse intensity in female and male *Cntnap2* KO rats, respectively (Figure 3D, WT F vs. KO F, Sidak’s multiple comparisons test, 105 dB SPL: *p* = 0.0303, Figure 3E, WT M vs. KO M, Sidak’s multiple comparisons test, 85–115 dB SPL: all *p* < 0.0001). While ASR magnitudes were similar in female and male *Cntnap2* WT rats (F WT: *n* = 6, M WT: *n* = 5, two-way ANOVA, intensity × sex *p* < 0.9551, *F*_(10,99)_ = 0.3743, intensity *p* < 0.0001, *F*_(10,99)_ = 59.17, sex* p* < 0.1054, *F*_(1,99)_ = 2.670), exaggerated ASR magnitudes were more pronounced in male than female *Cntnap2* KO rats (note the higher startle magnitudes in *Cntnap2* KO males compared with KO females in Figures 3E,D, respectively, F: *n* = 6, M: *n* = 5, two-way ANOVA, intensity × sex *p* < 0.0001, *F*_(10,99)_ = 6.864, intensity *p* < 0.0001, *F*_(10,99)_ = 103.9, sex* p* < 0.0001, *F*_(1,99)_ = 167.3).

To optimize the comparison of ASR magnitudes especially to moderate startle pulse intensities between animals (see hypothetical plasticity of ASR I-O in Figure 2 and Supplementary Figure 3), ASR magnitudes of all animals were normalized to their individual magnitude at the loudest startle pulse intensity (115 dB SPL). Normalization of ASR I-O magnitudes eliminated sex differences and the data were pooled for male and female *Cntnap2* WT or KO rats, respectively (Figure 3F, *Cntnap2* WT F: *n* = 6, M: *n* = 5, *Cntnap2* KO F: *n* = 6, M: *n* = 6, three-way ANOVA, intensity × genotype × sex *p* = 0.4238, *F*_(10,198)_ = 1.025, genotype × sex *p* = 0.1392, *F*_(1,198)_ = 2.205, intensity × sex *p* = 0.6617, *F*_(10,198)_ = 0.7657, intensity × genotype* p* < 0.0001, *F*_(10,198)_ = 5.021, sex* p* = 0.8462, *F*_(1,198)_ = 0.03771, genotype* p* < 0.0001, *F*_(1,198)_ = 21.53, intensity *p* < 0.0001, *F*_(10,198)_ = 307.1). Normalized startle magnitudes in *Cntnap2* KO rats were increased in comparison to *Cntnap2* WT rats, particularly at 90–100 dB SPL (Figure 3F, *Cntnap2* WT: *n* = 11, *Cntnap2* KO: *n* = 11, two-way ANOVA, intensity × genotype *p* < 0.0001, *F*_(10,220)_ = 4.750, intensity *p* < 0.0001, *F*_(10,220)_ = 313.5, genotype* p* < 0.0001, *F*_(1,220)_ = 20.63, Sidak’s multiple comparisons test, 90 dB SPL: *p* < 0.0001, 95 dB SPL: *p* = 0.0005, 100 dB SPL: *p* = 0.0069).

Besides the change in maximum startle response obtainable (ASR capacity, Figures 3D,E), the relationship between the startle pulse intensity and response magnitude could be altered in *Cntnap2* KO rats through several underlying mechanisms (Figure 2). To extract dynamic range characteristics including startle threshold and saturation from the startle I-O growth functions of individual animals, sigmoidal curves were fitted to the experimental data (scaled between 0 and 1). The ASR threshold was defined as 25%, and the ASR saturation as 90% of the scaled magnitude. The average fitted curves were significantly different between *Cntnap2* WT and KO rats, with fitted curves from KO rats showing both a steeper slope and a leftward shift of ES50 (startle pulse intensity that gives the half-maximal response, Figure 3G and Table 1, *Cntnap2* WT: *n* = 11 rats, *Cntnap2* KO: *n* = 11 rats, *p* < 0.0001). Increased startle magnitudes and altered dynamic range in *Cntnap2* KO rats (Figures 3D–G) were paralleled by a significantly lower startle threshold (Figure 3H Left, *Cntnap2* WT: 86.6 ± 4.29 dB SPL, KO: 82.7 ± 2.97 dB SPL, two-sided student’s *t*-test: *p* = 0.0210) and saturation (Figure 3H Right, *Cntnap2* WT: 106.5 ± 6.8 dB SPL, KO: 97.8.7 ± 3.6 dB SPL, two-sided student’s *t*-test: *p* = 0.0012). This means that, on average, *Cntnap2* KO rats reach the 25% and 90% criterion at lower startle pulse intensities than WT rats—further indicators for the left-shift of the ASR I-O function and increased acoustic reactivity in *Cntnap2* KO rats. Taken together, the ASR I-O functions and their parameters extracted from the sigmoidal curve fits demonstrated increased ASR capacity (maximal response possible), stimulus potency (ES50), ASR excitability (ASR threshold), dynamic range top plateau (ASR saturation), and ASR efficiency (slope) in *Cntnap2* KO rats.

**Table 1:** Comparison of sigmoidal curve fit of ASR I-O function with magnitude scaled between 0 and 1 in *Cntnap2* WT and KO rats corresponding to Figure 3G.

Best-fit values	*Cntnap2 WT*		*Cntnap2 KO*
Bottom	=0		=0
Top	=1		=1
ES50	92.64		87.49
HillSlope	14.55		18.41
Sy.x	0.1163		0.08851
Different curve fits?		<0.0001***	
Different slopes?		0.0088**	
Different ES50?		<0.0001***	

Bottom plateau constraint to 0, Top plateau constraint to 1, ES50: acoustic pulse intensity (dB SPL) that gives a startle magnitude halfway between Bottom and Top, HillSlope: steepness of the curve, Sy.x: standard error of regression, Different curves: curve fit comparison between *Cntnap2* WT and KO rats. *p* values, **p < 0.01, ***p < 0.001.

ASR magnitude and latency are in general negatively correlated (i.e., the higher the magnitude, the shorter the latency; Hoffman and Searle, 1968). Peak latencies in *Cntnap2* WT and KO rats were investigated across the ASR dynamic range, in particular at near startle I-O threshold, at ES50, and saturation (Figure 3I). Thereby, we could compare individual latencies at startle pulse intensities that yielded similar ASR magnitudes in *Cntnap2* WT and KO rats relative to their dynamic range. As expected, *Cntnap2* WT rats showed a negative relationship between startle pulse intensities across the dynamic range and peak startle latency (Figure 3I, slope *m* = −8.537 ms/increment, deviation from zero *p* = 0.0291, *F*_(1,1)_ = 479.3), indicating shortening of latency with increasing ASR magnitudes across the dynamic range. In *Cntnap2* KO rats, however, no such negative relationship was found (Figure 3I, slope *m* = 4.910 ms/increment, deviation from zero *p* = 0.3277, *F*_(1,1)_ = 3.126, WT vs. KO *p* = 0.0408). In contrast to the WT controls, *Cntnap2* KO rats showed significantly shorter latencies near startle I-O threshold (*Cntnap2* WT: 304.1 ± 22.3 ms, KO: 284.31 ± 12.2 ms, two-sided student’s *t*-test, *p* = 0.0181) and their startle peak latencies did not further decrease across the dynamic range (Figure 3I, deviation from zero *p* = 0.3277, *F*_(1,1)_ = 3.126). The shorter peak latencies near the threshold and the lack of further shortening of latency across the dynamic range are indicators for an overall increased response strength in *Cntnap2* KO rats. Taken together, our results show that *Cntnap2* KO rats have increased auditory reactivity and impaired habituation.

### Excitatory and Inhibitory Neurotransmitter Levels Are Altered in the Startle-Mediating Brainstem From *Cntnap2* KO Rats

**Figure 4:**
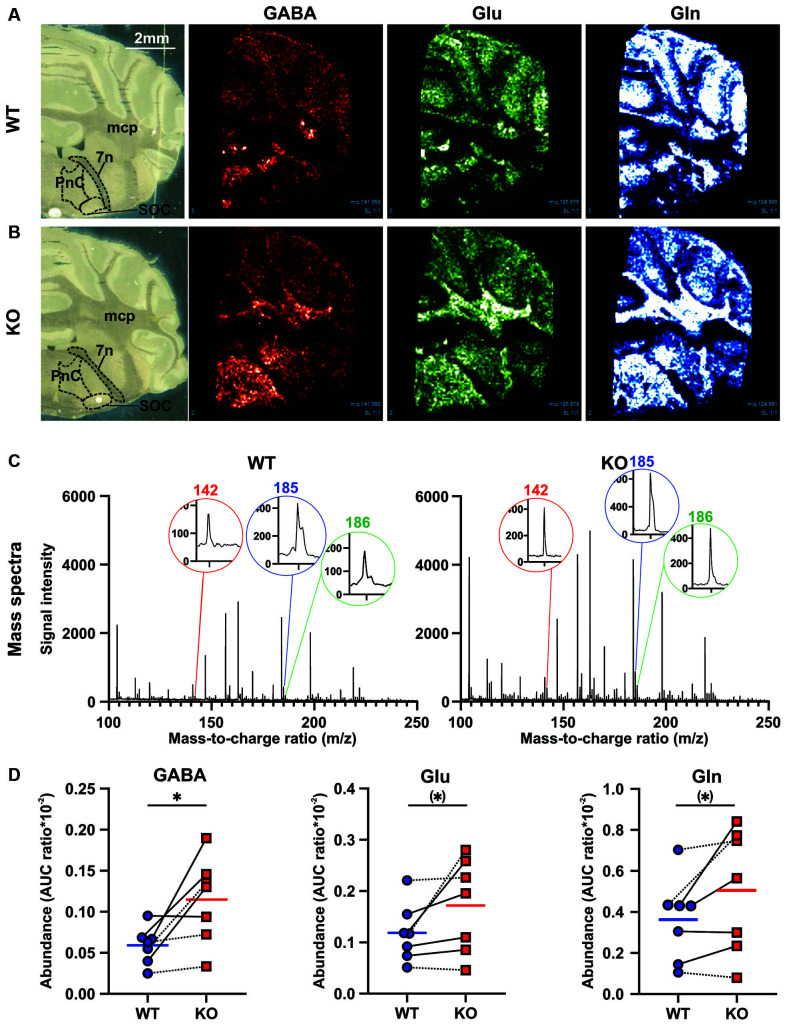
Effect of *Cntnap2* knockout on the MALDI MS signals of amino acids in fresh frozen rat brain tissue. MALDI-MS generated intensity maps of GABA ([GABA+K]^+^: 142m/z), glutamate ([Glu+K]^+^: 186 m/z), and glutamine ([Gln+ K]^+^: 185m/z) from a **(A)** *Cntnap2* WT and **(B)** KO rat. The signal of GABA, Glu, and Gln appears to be enhanced in the brainstem including and surrounding the PnC and in the mcp region in *Cntnap2* KO rats. **(C)** Mass spectra from the PnC region of interest acquired on a *Cntnap2* WT (**Left**) and KO (**Right**) rat coronal brain slice with ZnO in the mass region 100–250. Mass peaks corresponding to neurotransmitters ([GABA+K]^+^: 142m/z, [Glu+K]^+^: 186m/z, [Gln+ K]^+^: 185m/z) were acquired from each mass spectra. **(D)** The degree of signal enhancement in the PnC region can be seen through pairwise comparative under the curve analysis for *Cntnap2* KO rats (*n* = 7, red squares and horizontal line) compared with WT controls (*n* = 7, blue circles and horizontal line). The area under the curve (AUC) ratio was significantly enhanced for GABA (**Left**, paired *t*-test *p* = 0.0242), and by tendency for Glu (**Middle**, paired *t*-test *p* = 0.0858) and Gln (**Right**, paired *t*-test *p* = 0.0703). Dotted lines denote female, solid lines male WT-KO pairs. Abbreviations: PnC, nucleus reticularis pontis caudalis; mcp, middle cerebellar peduncle; 7n, facial nerve. Scale bar: 2mm. ^(*)^*p* < 0.1, *p < 0.05.

In order to assess possible alterations in neuronal excitation/inhibition within the startle-mediating brainstem circuitry that might underlie ASD-related sensory processing deficits (for review, see Sinclair et al., 2017b), we quantified GABA, glutamate, and glutamine amino acid levels ([GABA+K]^+^: 142m/z, [Glu+K]^+^: 186m/z, [Gln+K]^+^: 185m/z) in the PnC (nucleus reticularis pontis caudalis) of fresh frozen coronal brain tissue sections from adult *Cntnap2* WT and KO rats using MALDI MS (Figure 4, Table 2). Visual inspection of the intensity map images showed an increase in signal intensity of all three amino acids in the brainstem and middle cerebellar peduncle region of *Cntnap2* KO (Figure 4B) compared with WT rats (Figure 4A). The AUC analysis of individual amino acid peaks in the mass spectra of the PnC region (Figure 4C) showed a significant increase in AUC ratio for GABA (Figure 4D Left, WT *n* = 7, KO *n* = 7, paired *t*-test *p* = 0.0242). AUC ratios of glutamate (Figure 4D Middle, paired *t*-test *p* = 0.0858) and glutamine (Figure 4D Right, paired *t*-test *p* = 0.0703) were slightly increased by statistical tendency. Comparative analysis (Table 2) showed a 2-fold increase in GABA in the PnC region from *Cntnap2* KO rats (one sample *t*-test *p* = 0.0222) and by tendency a 1.4-fold increase in both glutamate (one sample *t*-test *p* = 0.0935) and glutamine (one sample *t*-test *p* = 0.0814). Consequently, the ratio between Glu/Gln was similar in *Cntnap2* KO and WT rats (one sample *t*-test *p* = 0.4051), whereas GABA/Gln was significantly enhanced (one sample *t*-test *p* = 0.0335, Table 2). Finally, GABA was more enhanced than Glu, as evidenced by significantly decreased Glu/GABA ratio (one sample *t*-test *p* = 0.0349) and a slight, yet statistically insignificant, increase in GABA/Glu ratio (one sample *t*-test *p* = 0.0853). Importantly, the comparative analysis showed no differences in two other metabolite levels in the PnC region (Table 2), i.e., [Choline+K]^+^: 143m/z (one sample *t*-test *p* = 0.3336) and [Norepinephrine+K]^+^: 208m/z (one sample *t*-test *p* = 0.1383), indicating that the increases in Glu, GABA, and Gln levels were not based on a general impairment in metabolism or neurotransmission in *Cntnap2* KO rats. Furthermore, GABA, Glu, and Gln levels were not altered in the SOC within the auditory brainstem of *Cntnap2* KO rats (Supplementary Figure 4). This indicated that the amino acid level increases in the PnC in *Cntnap2* KO rats were not ubiquitous throughout the brain. Taken together, our findings indicate aberrant levels of GABA, Glu, and Gln in the PnC of *Cntnap2* KO rats. This suggests that altered implicit auditory-evoked behaviors linked with functional deletion of *Cntnap2* are associated with an imbalance of excitation and inhibition, particularly affecting the GABA neurotransmitter system.

**Table 2:** Statistical comparison of MALDI MS AUC ratio (AUC ratio*10^−2^, paired *t*-test) and fold changes (one sample *t* test) for GABA, Glu, Gln, Choline, and Norepinephrine in the PnC region of experimentally naïve *Cntnap2* WT and KO rats.

	AUC ratio			Fold change								
Genotype	GABA	Glu	Gln	GABA	Glu	Gln	Glu/GABA	GABA/Glu	Glu/Gln	GABA/Gln	Choline	Norepinephrine
*Cntnap2* WT	0.059	0.118	0.363	1	1	1	1	1	1	1	1	1
*Cntnap2* KO	0.114	0.172	0.506	2.025	1.445	1.360	0.7671	1.442	1.066	1.487	1.108	1.485
*t*-test	0.0242*	0.0858^(*)^	0.0703^(*)^	0.0222*	0.0935^(*)^	0.0814^(*)^	0.0349*	0.0853^(*)^	0.4051^n.s.^	0.0335*	0.3336^n.s.^	0.1383^n.s.^

Post hoc t test: ^(*)^*p* < 0.1, *p < 0.05, n.s.: not significant.

### R-Baclofen Treatment Improves Disruptions in Habituation in *Cntnap2* KO Rats

**Figure 5:**
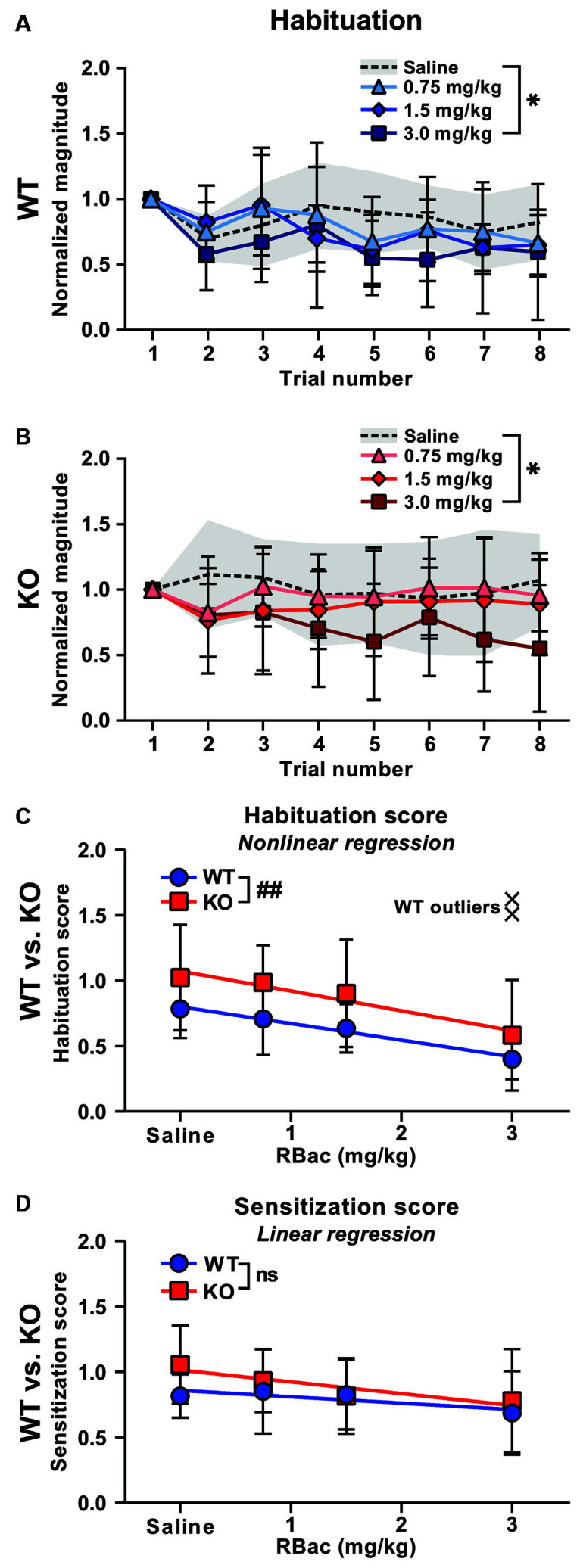
Higher doses of R-Baclofen (RBac) normalize habituation in *Cntnap2* KO rats. **(A,B)** Mean ± SD startle response magnitudes across eight subsequent trials normalized to the first trial in *Cntnap2* WT (**A**, blue symbols) and KO rats (**B**, red symbols) 1 h after injection of 0.75 mg/kg (triangles and error bars), 1.5 mg/kg (diamonds and error bars), or 3.0 mg/kg (squares and error bars) R-Baclofen compared with vehicle (saline, dotted line and gray area). Values <1 indicate habituation of the startle response. 3 mg/kg R-Baclofen led to a greater decline of startle magnitudes across the first eigth trials in comparison with saline administration in both *Cntnap2* WT (**A**, *n* = 11, Two-way RM ANOVA, trial *p* < 0.0001, *F*_(7,80)_ = 8.884, treatment *p* = 0.0071, *F*_(2.647, 211.7)_ = 4.397, trial × treatment *p* = 0.8352, *F*_(21,240)_ = 0.6965, Dunnett’s multiple comparisons test, saline vs. 0.75 mg/kg *p* = 0.6549, saline vs. 1.5 mg/kg *p* = 0.1267, saline vs. 3 mg/kg *p* = 0.0084) and *Cntnap2* KO rats (**B**, *n* = 11, Two-way RM ANOVA, trial *p* = 0.6752, *F*_(7,80)_ = 0.6960, treatment *p* < 0.0001, *F*_(2.864, 229.1)_ = 10.16, trial × treatment *p* = 0.7925, *F*_(21,240)_ = 0.7925, Dunnett’s multiple comparisons test, saline vs. 0.75 mg/kg *p* = 0.7085, saline vs. 1.5 mg/kg *p* = 0.0606, saline vs. 3 mg/kg *p* < 0.0001). **(C)** Straight-line regression of the habituation scores in *Cntnap2* WT (blue circles and error bars, mean ± SD) and KO rats (red squares and error bars, mean ± SD). Mixed-effects analysis showed significantly reduced habituation scores with 3 mg/kg R-Baclofen in comparison to saline in both genotypes (*Cntnap2* WT: *n* = 9–11 rats, *Cntnap2* KO: *n* = 11 rats, genotype *p* = 0.0034, *F*_(1,20)_ = 11.03, treatment *p* = 0.0005, *F*_(2.785, 53.84)_ = 7.327, treatment × genotype *p* = 0.9632, *F*_(3,58)_ = 0.09379, Dunnett’s multiple comparison’s test, WT: saline vs. 0.75 mg/kg *p* = 0.8601, saline vs. 1.5 mg/kg *p* = 0.2785, saline vs. 3 mg/kg: *p* = 0.0118; KO: saline vs. 0.75 mg/kg *p* = 0.9861, saline vs. 1.5 mg/kg *p* = 0.8595, saline vs. 3 mg/kg: *p* = 0.0205). The slopes of the regression lines showed no R-Baclofen dose-dependent differences in *Cntnap2* WT (*n* = 9–11 rats, Y = −0.1273 * X + 0.8004, Sy.*x* = 0.2131) and KO rats (*n* = 11 rats, Y = −0.1514* X + 1.073, Sy.*x* = 0.3777), whereas the elevations of the regression lines were significantly different (*p* = 0.0093). Two *Cntnap2* WT outliers at 3 mg/kg R-Baclofen (black crosses) were excluded from the straight-line regression and mixed-effects analysis using Prism GraphPad’s “Detect and eliminate outliers” method. **(D)** R-Baclofen treatment did not induce a statistically significant reduction in sensitization scores, neither in *Cntnap2* WT nor KO rats (two-way RM-ANOVA, *Cntnap2* WT: *n* = 11 rats, *Cntnap2* KO: *n* = 11 rats, genotype *p* = 0.0966, *F*_(1,20)_ = 3.040, treatment *p* = 0.1430, *F*_(2.628, 52.56)_ = 1.930, treatment × genotype *p* = 0.5867, *F*_(3,60)_ = 0.6490). Regression lines were similar in both genotypes (*Cntnap2* WT: Y = −0.04851 * X + 0.8589, Sy.*x* = 0.05019, *Cntnap2* KO: Y = −0.08989 * X + 1.014, Sy.*x* = 0.05991, WT vs. KO: slopes *p* = 0.3053, elevation *p* = 0.0548). *p < 0.05; ^##^*p* < 0.01 (comparison of regression lines); n.s.: not significant.

We first investigated the potential of R-Baclofen to remediate perturbed short-term habituation in *Cntnap2* KO rats. Short-term habituation of the startle response was measured across the first eight startle trials of the test day 1 h after systemic injection of 0.75, 1.5, or 3 mg/kg R-Baclofen (Figure 5). In both *Cntnap2* WT (Figure 5A) and KO rats (Figure 5B), the highest dose of R-Baclofen at 3 mg/kg led to a greater decline of startle magnitudes across the first eight trials in comparison with saline administration (Figure 5A, *Cntnap2* WT: *n* = 11, Two-way RM ANOVA, trial *p* < 0.0001, *F*_(7,80)_ = 8.884, treatment *p* = 0.0071, *F*_(2.647, 211.7)_ = 4.397, trial × treatment *p* = 0.8352, *F*_(21,240)_ = 0.6965, Dunnett’s multiple comparisons test, saline vs. 0.75 mg/kg *p* = 0.6549, saline vs. 1.5 mg/kg *p* = 0.1267, saline vs. 3 mg/kg *p* = 0.0084, Figure 5B, *Cntnap2* KO: *n* = 11, Two-way RM ANOVA, trial *p* = 0.6752, *F*_(7,80)_ = 0.6960, treatment *p* < 0.0001, *F*_(2.864, 229.1)_ = 10.16, trial × treatment *p* = 0.7925, *F*_(21,240)_ = 0.7925, Dunnett’s multiple comparisons test, saline vs. 0.75 mg/kg *p* = 0.7085, saline vs. 1.5 mg/kg *p* = 0.0606, saline vs. 3 mg/kg *p* < 0.0001). Habituation scores (Figure 5C) and sensitization scores (Figure 5D) were calculated and compared across R-Baclofen doses within genotype, and between equally treated *Cntnap2* WT and KO rats. Mixed-effects analysis showed significantly reduced habituation scores with 3 mg/kg R-Baclofen in comparison to saline in both genotypes, thereby confirming enhanced short-term habituation through R-Baclofen in *Cntnap2* WT and KO rats (Figure 5C, Mixed-effects analysis, *Cntnap2* WT: *n* = 9–11 rats, *Cntnap2* KO: *n* = 11 rats, genotype *p* = 0.0034, *F*_(1,20)_ = 11.03, treatment *p* = 0.0005, *F*_(2.785, 53.84)_ = 7.327, treatment × genotype *p* = 0.9632, *F*_(3,58)_ = 0.09379, Dunnett’s multiple comparison’s test, WT: saline vs. 0.75 mg/kg *p* = 0.8601, saline vs. 1.5 mg/kg *p* = 0.2785, saline vs. 3 mg/kg: *p* = 0.0118; KO: saline vs. 0.75 mg/kg *p* = 0.9861, saline vs. 1.5 mg/kg *p* = 0.8595, saline vs. 3 mg/kg: *p* = 0.0205). To further analyze the effects of the three doses of R-Baclofen on short-term habituation between *Cntnap2* WT and KO rats, we performed straight-line regressions of the habituation scores depending on the treatment, and compared the slopes and elevations of the two regression lines (Figure 5C). The elevations of the regression lines were significantly different in *Cntnap2* KO compared with WT rats, resulting from the overall greater habituation scores across treatments in *Cntnap2* KO rats (Figure 5C, *Cntnap2* WT: *n* = 9–11 rats, elevation: *c* = 0.8004, *Cntnap2* KO: *n* = 11 rats, elevation: *c* = 1.073, *p* = 0.0093). The slopes were similar in *Cntnap2* WT and KO rats, showing a negative relationship between the R-Baclofen dose and habituation score in both genotypes (i.e., the higher the dose, the lower the habituation score, Figure 5C, *Cntnap2* WT: *n* = 9–11 rats, slope: *m* = −0.1273, *Cntnap2* KO: *n* = 11 rats, slope: *m* = −0.1514, *p* = 0.6940). This indicates that the selective activation of GABA_B_ receptors by R-Baclofen had a similar suppressive mode of action on habituation scores in *Cntnap2* WT and KO rats. In contrast to short-term habituation, R-Baclofen did not induce a statistically significant reduction in sensitization scores, neither within nor between *Cntnap2* WT and KO rats (Figure 5D, two-way RM-ANOVA, *Cntnap2* WT: *n* = 11 rats, *Cntnap2* KO: *n* = 11 rats, genotype *p* = 0.0966, *F*_(1,20)_ = 3.040, treatment *p* = 0.1430, *F*_(2.628, 52.56)_ = 1.930, treatment × genotype *p* = 0.5867, *F*_(3,60)_ = 0.6490; Linear regression, *Cntnap2* WT: Y = −0.04851 * X + 0.8589, Sy.*x* = 0.05019, *Cntnap2* KO: Y = −0.08989 * X + 1.014, Sy.*x* = 0.05991, WT vs. KO: slopes *p* = 0.3053, elevation *p* = 0.0548). Taken together, our results suggest that higher doses of R-Baclofen have the potential to improve deficient sensory filtering in *Cntnap2* KO rats by enhancing short-term habituation. Sensitization of the ASR, however, appeared insensitive to the influence of R-Baclofen. This indicates that the cellular mechanisms or neural circuits controlling short-term habituation and sensitization are not affected the same way by selective activation of GABA_B_ receptors though systemic administration of R-Baclofen.

### R-Baclofen Ameliorates Exaggerated ASRs in *Cntnap2* KO Rats to Moderate, but Not to High Startle Pulse Intensities

**Figure 6:**
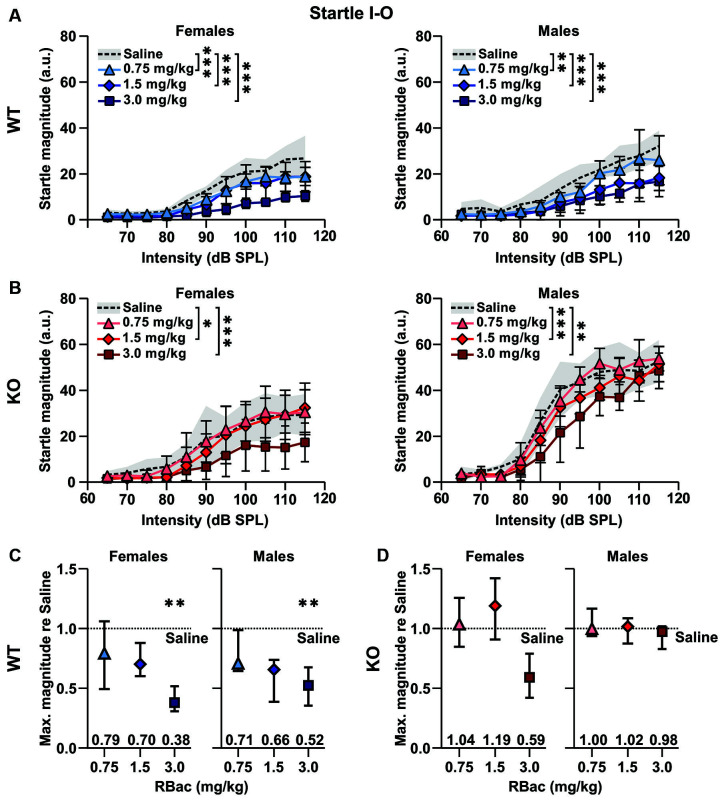
Medium and high doses of R-Baclofen decrease ASR magnitudes in *Cntnap2* KO rats to control levels. **(A,B)** Mean ± SD startle response magnitudes to increasing startle pulse intensities after injection of saline (dotted line and gray area), 0.75 mg/kg (triangles), 1.5 mg/kg (diamonds), 3 mg/kg R-Baclofen (squares) in *Cntnap2* WT rats (**A**, blue symbols) and *Cntnap2* KO rats (**B**, red symbols). Startle magnitudes were significantly reduced after injection of 0.75, 1.5, or 3 mg/kg R-Baclofen in comparison with injection of saline in female **(A, Left)** and male **(A, Right)** *Cntnap2* WT rats (**A, Left**, *Cntnap2* WT F: *n* = 6, two-way RM ANOVA, intensity *p* < 0.0001, *F*_(10,55)_ = 44.28, treatment *p* < 0.0001, *F*_(2.453, 134.9)_ = 86.07, intensity × treatment *p* < 0.0001, *F*_(30,165)_ = 4.390, Dunnett’s multiple comparisons test, saline vs. 0.75 mg/kg *p* < 0.0001, saline vs. 1.5 mg/kg *p* < 0.0001, saline vs. 3 mg/kg: *p* < 0.0001; **A, Right**, *Cntnap2* WT M: *n* = 5, two-way RM ANOVA, intensity *p* < 0.0001, *F*_(10,44)_ = 21.39, treatment *p* < 0.0001, *F*_(2.511, 110.5)_ = 63.83, intensity × treatment *p* < 0.0001, *F*_(30,132)_ = 2.847, Dunnett’s multiple comparisons test, saline vs. 0.75 mg/kg *p* = 0.0002, saline vs. 1.5 mg/kg *p* < 0.0001, saline vs. 3 mg/kg: *p* < 0.0001). **(B)** Startle magnitudes in female **(B, Left)** and male **(B, Right)** *Cntnap2* KO rats were significantly reduced after injection of 1.5, or 3 mg/kg, but not with 0.75 mg/kg, R-Baclofen in comparison with saline injection (**B, Left**, *Cntnap2* KO F: *n* = 6, Two-way RM ANOVA, intensity *p* < 0.0001, *F*_(10,55)_ = 17.99, treatment *p* < 0.0001, *F*_(1.911, 105.1)_ = 29.81, intensity × treatment *p* = 0.0405, *F*_(30,165)_ = 1.568, Dunnett’s multiple comparisons test, saline vs. 0.75 mg/kg *p* > 0.9999, saline vs. 1.5 mg/kg *p* = 0.0309, saline vs. 3 mg/kg: *p* < 0.0001; **B, Right**, *Cntnap2* KO M: *n* = 5, Two-way RM ANOVA, intensity *p* < 0.0001, *F*_(10,44)_ = 77.47, treatment *p* < 0.0001, *F*_(2.137, 94.04)_ = 20.74, intensity × treatment *p =* 0.1091, *F*_(30,132)_ = 1.384, Dunnett’s multiple comparisons test, saline vs. 0.75 mg/kg *p* = 0.9802, saline vs. 1.5 mg/kg *p* = 0.0003, saline vs. 3 mg/kg: *p* < 0.0001). **(C,D)** Comparison of ASR maximum response. **(C)** In both female (**Left**) and male (**Right**) *Cntnap2* WT rats, R-Baclofen induced a significant decrease in the maximum ASR capacity at 115 dB SPL (WT F: Friedman test, *p* = 0.0085, Dunn’s multiple comparisons test, 0.75 mg/kg *p* > 0.9999, 1.5 mg/kg *p* = 0.5391, 3.0 mg/kg *p* = 0.0052; WT M: Friedman test, *p* = 0.0120, Dunn’s multiple comparisons test, 0.75 mg/kg *p* = 0.6620, 1.5 mg/kg *p* = 0.0825, 3.0 mg/kg *p* = 0.0099). **(D)** R-Baclofen did not induce a decrease in maximum ASR capacity from female (**Left**) nor male (**Right**) *Cntnap2* KO rats (KO F: Friedman test, *p* = 0.1268; KO M: Friedman test, *p* = 0.3720). *p < 0.05; **p < 0.01; ***p < 0.001.

The relationship between startle pulse intensities and ASR magnitudes can be altered through a number of variables, such as the genotype and pharmaceuticals (Figure 2; for review, see Koch, 1999). We next aimed to test if R-Baclofen could decrease the enhanced ASR magnitudes and ASR capacity in *Cntnap2* KO rats. We first compared the effects of R-Baclofen on the ASR I-O function and maximal response magnitudes within genotype and sex (Figure 6). In *Cntnap2* WT rats, all three doses of R-Baclofen (0.75, 1.5, 3 mg/kg) significantly decreased the ASR magnitudes to startle pulses of increasing intensity compared with saline in both females and males (Figure 6A Left, *Cntnap2* WT F: *n* = 6, Two-way RM ANOVA, intensity *p* < 0.0001, *F*_(10,55)_ = 44.28, treatment *p* < 0.0001, *F*_(2.453, 134.9)_ = 86.07, intensity × treatment *p* < 0.0001, *F*_(30,165)_ = 4.390, Dunnett’s multiple comparisons test, saline vs. 0.75 mg/kg *p* < 0.0001, saline vs. 1.5 mg/kg *p* < 0.0001, saline vs. 3 mg/kg: *p* < 0.0001; Right, *Cntnap2* WT M: *n* = 5, Two-way RM ANOVA, intensity *p* < 0.0001, *F*_(10,44)_ = 21.39, treatment *p* < 0.0001, *F*_(2.511, 110.5)_ = 63.83, intensity × treatment *p* < 0.0001, *F*_(30,132)_ = 2.847, Dunnett’s multiple comparisons test, saline vs. 0.75 mg/kg *p* = 0.0002, saline vs. 1.5 mg/kg *p* < 0.0001, saline vs. 3 mg/kg: *p* < 0.0001). This decrease in ASR magnitudes was evident across a wide range of startle pulse intensities after injection of 1.5 and 3 mg/kg R-Baclofen in female and male *Cntnap2* WT rats (*post hoc* comparisons matched for startle pulse intensities see Supplementary Table 1). In *Cntnap2* KO rats, 1.5 and 3 mg/kg R-Baclofen significantly reduced ASR magnitudes in both females and males, whereas the lowest dose of R-Baclofen (0.75 mg/kg) did not (Figure 6B Left, *Cntnap2* KO F: *n* = 6, Two-way RM ANOVA, intensity *p* < 0.0001, *F*_(10,55)_ = 17.99, treatment *p* < 0.0001, *F*_(1.911, 105.1)_ = 29.81, intensity × treatment *p* = 0.0405, *F*_(30,165)_ = 1.568, Dunnett’s multiple comparisons test, saline vs. 0.75 mg/kg *p* > 0.9999, saline vs. 1.5 mg/kg *p* = 0.0309, saline vs. 3 mg/kg: *p* < 0.0001; Right, *Cntnap2* KO M: *n* = 5, Two-way RM ANOVA, intensity *p* < 0.0001, *F*_(10,44)_ = 77.47, treatment *p* < 0.0001, *F*_(2.137, 94.04)_ = 20.74, intensity × treatment *p =* 0.1091, *F*_(30,132)_ = 1.384, Dunnett’s multiple comparisons test, saline vs. 0.75 mg/kg *p* = 0.9802, saline vs. 1.5 mg/kg *p* = 0.0003, saline vs. 3 mg/kg: *p* < 0.0001). Interestingly, in both female and male *Cntnap2* KO rats, the reduction in ASR magnitude was only present in response to weaker, but not to higher startle pulse intensities (*post hoc* comparisons matched for startle pulse intensities see Supplementary Table 1). To further investigate the effect of R-Baclofen on maximum ASR capacity, we compared the ASR magnitudes at the loudest startle pulse (115 dB SPL) relative to respective saline controls (Figures 6C,D). In female and male *Cntnap2* WT rats, R-Baclofen induced a dose-dependent reduction in maximal ASR magnitude [Figure 6C, median (IQR), WT F: *n* = 6 rats, 0.75 mg/kg re saline: 0.79 (0.49–1.06), 1.5 mg/kg re saline: 0.70 (0.60–0.88), 3 mg/kg re saline: 0.38 (0.30–0.52), Friedman test, *p* = 0.0085, Dunn’s multiple comparisons test, 0.75 mg/kg vs. saline: *p* > 0.9999, 1.5 mg/kg vs. saline: *p* = 0.5391, 3 mg/kg vs. saline: *p* = 0.0052; WT M: *n* = 5 rats, 0.75 mg/kg re saline: 0.71 (0.64–0.99), 1.5 mg/kg re saline: 0.66 (0.39–0.74), 3 mg/kg re saline: 0.52 (0.35–0.68), Friedman test, *p* = 0.0120, Dunn’s multiple comparisons test, 0.75 mg/kg vs. saline: *p* = 0.6620, 1.5 mg/kg vs. saline: *p* = 0.0825, 3.0 mg/kg vs. saline: *p* = 0.0099]. In contrast, maximal ASR magnitudes were similar irrespective of treatment in both female and male *Cntnap2* KO rats (Figure 6D, median (IQR), KO F: *n* = 6 rats, 0.75 mg/kg re saline: 1.04 (0.85–1.26), 1.5 mg/kg re saline: 1.19 (0.91–1.42), 3 mg/kg re saline: 0.59 (0.42–0.79), Friedman test, *p* = 0.1268; KO M: *n* = 5 rats, 0.75 mg/kg re saline: 1.00 (0.94–1.17), 1.5 mg/kg re saline: 1.02 (0.88–1.09), 3 mg/kg re saline: 0.98 (0.83–1.02), Friedman test, *p* = 0.3720). In summary, as expected, R-Baclofen decreased magnitudes in the ASR I-O growth functions. In doing so, *Cntnap2* KO rats showed a higher minimal effective dose (1.5 mg/kg) than WT rats (0.75 mg/kg). The reduction in ASR magnitudes in *Cntnap2* KO rats was restricted to lower startle pulse intensities, whereas the increased maximal ASR capacity was not ameliorated by R-Baclofen. This notion was further corroborated by between genotype comparisons, in particular, ASR magnitudes in *Cntnap2* KO males after R-Baclofen treatment compared to saline-injected WT males (Supplementary Figures 5D–F). R-Baclofen dose-dependently reduced ASR magnitudes in *Cntnap2* KO males and brought them closer to WT control levels. However, ASR magnitudes were most notably downregulated for low to medium startle pulse intensities, but not for the highest startle pulse intensities tested (Supplementary Figures 5D–F). Furthermore, *post hoc* testing of normalized ASR I-O functions matched for startle pulse intensities did not find statistically significant differences between treatments in *Cntnap2* WT rats, whereas in *Cntnap2* KO rats ASR magnitudes were reduced in particular at 85 and 90 dB SPL after 1.5 mg/kg R-Baclofen and at 90 dB SPL after 3 mg/kg R-Baclofen administration (Dunnett’s multiple comparisons test, saline vs. 1.5 mg/kg: 85 dB SPL: *p* = 0.0105, 90 dB SPL: *p* = 0.0322; saline vs. 3 mg/kg: 90 dB SPL: *p* = 0.0375, Supplementary Figure 6). The minimal effective dose of R-Baclofen in *Cntnap2* KO rats determined from their normalized ASR magnitudes after treatment with R-Baclofen compared to those in WT rats after saline injection was 1.5 mg/kg (Supplementary Figures 6C–E). These differences between *Cntnap2* WT and KO rats after normalizing magnitudes to the individual ASR capacities further emphasize the differential effect of R-Baclofen on ASR I-O growth functions in the two genotypes. It suggests that the R-Baclofen effect was distinctly suppressive on ASRs to weaker startle pulse intensities in KO rats. In contrast, lack of such a suppression indicated that in WT rats R-Baclofen particularly impacted their ASRs to higher startle pulse intensities.

### R-Baclofen Treatment Normalizes ASR I-O Threshold and Saturation in *Cntnap2* KO Rats, but Exacerbates Shorter ASR Peak Latencies

**Figure 7:**
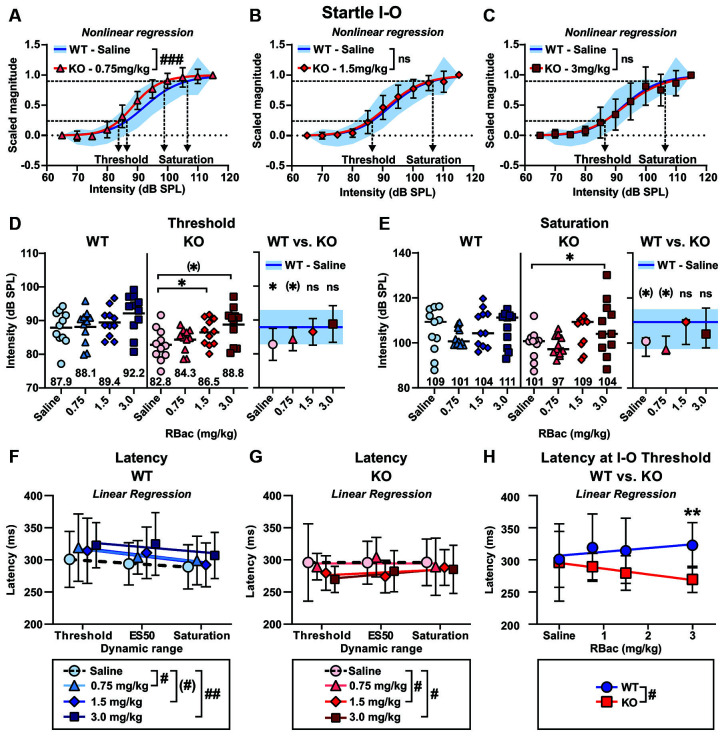
Medium and high doses of R-Baclofen increase ASR I-O threshold and saturation in *Cntnap2* KO rats close to controls but shorten ASR peak latencies. **(A–C)** Sigmoidal curves (lines) fitted to the startle magnitudes scaled between 0 and 1 in *Cntnap2* WT rats with saline (SD, blue area) and *Cntnap2* KO rats (mean ± SD) with 0.75 mg/kg (**A**, red triangles and error bars), 1.5 mg/kg (**B**, red diamonds and error bars), and 3 mg/kg R-Baclofen (**C**, red squares and error bars). Dotted horizontal line at 0.25 determined as ASR threshold and at 0.9 as ASR saturation (curve fit values see Table 3). The average curve fit was significantly different from controls in *Cntnap2* KO rats after administration of 0.75 mg/kg (**A**, *p* < 0.0001, *F*_(2,238)_ = 12.12), but similar to controls with 1.5 mg/kg (**B**, *p* = 0.6048, *F*_(2,238)_ = 0.5039), and 3 mg/kg R-Baclofen (**C**, *p* = 0.7751, *F*_(2,238)_ = 0.2550). **(D)** Individual ASR thresholds in *Cntnap2* WT (**Left**), KO (**Middle**), and WT vs. KO rats (**Right**) extracted from individual sigmoidal curve fits (ASR threshold values see Supplementary Table 2). **(D, Left)** Mean ASR thresholds were not significantly increased in *Cntnap2* WT rats with R-Baclofen (0.75 mg/kg: light blue triangles and horizontal black line, 1.5 mg/kg: blue diamonds and horizontal black line, 3 mg/kg: dark blue squares and horizontal black line) compared to saline (circles and horizontal black line, RM ANOVA, *p* = 0.0685). **(D, Middle)** Mean ASR thresholds were significantly increased in *Cntnap2* KO rats with R-Baclofen compared to saline (saline: circles and horizontal black line, 0.75 mg/kg: light red triangles and horizontal black line, 1.5 mg/kg: red diamonds and horizontal black line, 3 mg/kg: dark red squares and horizontal black line, RM ANOVA, *p* = 0.0291, Dunnett’s multiple comparisons test, saline vs. 0.75 mg/kg: *p* = 0.5583, saline vs. 1.5 mg/kg: *p* = 0.0315, saline vs. 3 mg/kg: *p* = 0.0784). **(D, Right)** Mean ± SD ASR thresholds in *Cntnap2* KO rats were significantly different from WT controls (after saline, blue line and area) with saline (two-sided student’s *t*-test, *p* = 0.0244), not quite significantly different with 0.75 mg/kg (two-sided student’s *t*-test, *p* = 0.0690), and similar to controls with 1.5 mg/kg (two-sided student’s *t*-test, *p* = 0.4839) and 3 mg/kg R-Baclofen (two-sided student’s *t*-test, *p* = 0.6819). **(E)** Individual ASR saturation levels in *Cntnap2* WT (**Left**), KO (**Middle**), and WT vs. KO rats (**Right**) extracted from individual sigmoidal curve fits (ASR saturation values see Supplementary Table 2). **(E, Left)** Median ASR saturation levels were not significantly altered in *Cntnap2* WT rats with R-Baclofen (0.75 mg/kg: light blue triangles and horizontal black line, 1.5 mg/kg: blue diamonds and horizontal black line, 3 mg/kg: dark blue squares and horizontal black line) compared to saline (circles and horizontal black line, Friedman test, *p* = 0.5915). **(E, Middle)** Median ASR saturation levels were significantly increased in *Cntnap2* KO rats with R-Baclofen compared to saline (saline: circles and horizontal black line, 0.75 mg/kg: light red triangles and horizontal black line, 1.5 mg/kg: red diamonds and horizontal black line, 3 mg/kg: dark red squares and horizontal black line, Friedman test, *p* = 0.0240, Dunn’s multiple comparisons test, saline vs. 0.75 mg/kg: *p* > 0.9999, saline vs. 1.5 mg/kg: *p* = 0.2959, saline vs. 3 mg/kg: *p* = 0.0150). **(E, Right)** Median ± interquartile range (IQR) ASR saturation levels in *Cntnap2* KO rats were by tendency different from WT controls (after saline, blue line and area) with saline (Mann–Whitney test, *p* = 0.0879) and 0.75 mg/kg (Mann–Whitney test, *p* = 0.0879), and similar to controls with 1.5 mg/kg (Mann–Whitney test, *p* = 0.7477) and 3 mg/kg R-Baclofen (Mann–Whitney test, *p* = 0.8470). **(F,G)** Linear regression of ASR peak latencies across the dynamic range of *Cntnap2* WT **(F)** and KO **(G)** rats with saline or R-Baclofen (mean ± SD, linear regression fits see Table 4). Elevations of the regression lines were significantly different in *Cntnap2* WT (**F**, *p* = 0.0034, *F*_(3,7)_ = 12.46; saline vs. 0.75 mg/kg: *p* = 0.0202, *F*_(1,3)_ = 20.50; saline vs. 1.5 mg/kg: *p* = 0.0620, *F*_(1,3)_ = 8.468; saline vs. 3 mg/kg: *p* = 0.0099, *F*_(1,3)_ = 34.45) and KO rats **(G**; *p* = 0.0336, *F*_(3,7)_ = 5.192; saline vs. 0.75 mg/kg: *p* = 0.8075, *F*_(1,3)_ = 0.07070; saline vs. 1.5 mg/kg: *p* = 0.0375, *F*_(1,3)_ = 12.76; saline vs. 3 mg/kg: *p* = 0.0272, *F*_(1,3)_ = 16.37). **(H)** Linear regression of ASR peak latencies near threshold across treatment in *Cntnap2* WT (blue circles and error bars, mean ± SD) and KO rats (red squares and error bars, mean ± SD). Slopes of the regression lines were significantly different (*p* = 0.0116, *F*_(1,4)_ = 19.41; WT: blue line, *Y* = 5.893 * X + 306.6, Sy.*x* = 7.147; KO: red line, Y = −8.876 * X + 295.3, Sy.*x* = 2.057). ^(*)^*p* < 0.1; *p < 0.05; **p < 0.01; comparison of regression lines: ^(#)^*p* < 0.1; ^#^*p* < 0.05; ^##^*p* < 0.01; ^###^*p* < 0.001, n.s., not significant.

Sigmoidal curves were fitted to the ASR I-O data scaled between 0 and 1 for individual animals of both genotypes and all treatments. Average curve fits were significantly different between *Cntnap2* KO rats treated with 0.75 mg/kg R-Baclofen and *Cntnap2* WT rats after saline injection (Figure 7A and Table 3, *p* < 0.0001, *F*_(2,238)_ = 12.12). In contrast to this, average curve fits were similar between *Cntnap2* KO rats treated with 1.5 mg/kg (Figure 7B and Table 3, *p* = 0.6048, *F*_(2,238)_ = 0.5039) or 3 mg/kg R-Baclofen (Figure 7C and Table 3, *p* = 0.7751, *F*_(2,238)_ = 0.2550) compared with *Cntnap2* WT rats after saline injection. ASR thresholds extracted at the 25% scaled magnitude generally increased with the dose of R-Baclofen (Figure 7D and Supplementary Table 2). This increase was significant in *Cntnap2* KO rats in particular with 1.5 mg/kg R-Baclofen in comparison with saline, but not in WT rats (Figure 7D Left, *Cntnap2* WT rats (*n* = 11), mean ± SD saline: 87.9 ± 5.1 dB SPL, 0.75 mg/kg: 88.1 ± 5.0 dB SPL, 1.5 mg/kg: 89.4 ± 4.3 dB SPL, 3 mg/kg: 92.2 ± 5.8 dB SPL, RM ANOVA, *p* = 0.0685, Figure 7D Middle, *Cntnap2* KO rats (*n* = 11), saline: 82.8 ± 4.7 dB SPL, 0.75 mg/kg: 84.3 ± 3.4 dB SPL, 1.5 mg/kg: 86.5 ± 3.9 dB SPL, 3 mg/kg: 88.8 ± 5.4 dB SPL, RM ANOVA, *p* = 0.0291, Dunnett’s multiple comparisons test, saline vs. 0.75 mg/kg *p* = 0.5583, saline vs. 1.5 mg/kg *p* = 0.0315, saline vs. 3 mg/kg: *p* = 0.0784). Comparison of ASR thresholds in R-Baclofen-treated *Cntnap2* KO rats with saline-treated WT rats showed that thresholds were increased to control level after injection of 1.5 and 3 mg/kg R-Baclofen, while they were by tendency still lower than in controls with 0.75 mg/kg R-Baclofen (Figure 7D Right, two-sided student’s *t*-test, WT—Saline vs. KO–0.75 mg/kg: *p* = 0.0690, WT—Saline vs. KO–1.5 mg/kg: *p* = 0.4839, WT—Saline vs. KO–3 mg/kg: *p* = 0.6819). Saturation of the ASR I-O function extracted at the 90% scaled magnitude was significantly altered through R-Baclofen in *Cntnap2* KO rats, but not in WT rats (Figure 7E and Supplementary Table 2). In particular, 3 mg/kg R-Baclofen increased ASR I-O saturation in *Cntnap2* KO rats compared with saline [Figure 7E Left, *Cntnap2* WT rats (*n* = 11), median (IQR), saline: 109.3 (97.4-115.0) dB SPL, 0.75 mg/kg: 100.7 (99.1-106.7) dB SPL, 1.5 mg/kg: 104.3 (98.5-112.8) dB SPL, 3 mg/kg: 111.3 (97.7-112.2) dB SPL, Friedman test, *p* = 0.5915, Figure 7E Middle, *Cntnap2* KO rats (*n* = 11), saline: 100.8 (94.2-102.3) dB SPL, 0.75 mg/kg: 97.2 (95.3-103.1) dB SPL, 1.5 mg/kg: 109.3 (99.4-110.2) dB SPL, 3 mg/kg: 104.0 (97.9-115.5) dB SPL, Friedman test, *p* = 0.0240, Dunn’s multiple comparisons test, saline vs. 0.75 mg/kg: *p* > 0.9999, saline vs. 1.5 mg/kg: *p* = 0.2959, saline vs. 3 mg/kg: *p* = 0.0150]. Comparison between genotypes showed that ASR I-O saturation in *Cntnap2* KO rats with 1.5 and 3 mg/kg R-Baclofen was similar to saturation in WT rats after saline injection, while there was a slight, yet not quite significant, difference with 0.75 mg/kg (Figure 7E Right, Mann–Whitney test, WT—Saline vs. KO–0.75 mg/kg: *p* = 0.0879, WT—Saline vs. KO–1.5 mg/kg: *p* = 0.7477, WT—Saline vs. KO–3 mg/kg: *p* = 0.8470). Taken together, our results suggest that selective activation of GABA_B_ receptors by 1.5 mg/kg and 3 mg/kg R-Baclofen can normalize acoustic reactivity in *Cntnap2* KO rats through a right-shift in ASR I-O function and an increase in ASR threshold and saturation sound levels to control levels.

**Table 3:** Comparison of sigmoidal regression fit of ASR I-O function with magnitude scaled between 0 and 1 in *Cntnap2* WT and KO rats.

	RBac (mg/kg)	Saline	0.75	1.5	3
*Cntnap2 WT*	Bottom	=0	=0	=0	=0
	Top	=1	=1	=1	=1
	ES50	92.92	92.87	94.10	96.67
	HillSlope	15.92	19.96	17.41	16.52
	Sy.x	0.1978	0.1400	15.06	0.1809
*Cntnap2 KO*	Bottom	=0	=0	=0	=0
	Top	=1	=1	=1	=1
	ES50	87.96	88.82	91.97	93.47
	HillSlope	16.31	19.85	15.70	14.60
	Sy.x	0.1688**	0.1184**	0.1304^n.s.^	0.1999^n.s.^
KO vs. WT—Saline	Different curve fits?	<0.0001***	<0.0001***	0.6048^n.s.^	0.7751^n.s.^
	Different slopes?	0.8760^n.s.^	0.1152^n.s.^	0.9173^n.s.^	0.5915^n.s.^
	Different ES50?	<0.0001***	<0.0001***	0.3218^n.s.^	0.6345^n.s.^

Bottom plateau constraint to 0, Top plateau constraint to 1, ES50: acoustic pulse intensity (dB SPL) that gives a startle magnitude halfway between Bottom and Top, HillSlope: steepness of the curve, Sy.x: standard error of regression, KO vs. WT—Saline: curve fit comparison between *Cntnap2* KO rats treated with saline, 0.75, 1.5, or 3 mg/kg R-Baclofen and WT rats with saline. *p* values, ***p < 0.001, n.s.: not significant.

We next investigated R-Baclofen-related changes in ASR peak latencies in *Cntnap2* WT and KO rats across the ASR dynamic range (Figures 7F–H and Table 4). In *Cntnap2* WT rats, R-Baclofen increased the ASR peak latencies across the dynamic range by means of greater regression line elevations in comparison with saline (most notably at 0.75 and 3 mg/kg, Figure 7F and Table 4, elevation: *p* = 0.0034). In contrast to this, R-Baclofen decreased the ASR peak latencies across the dynamic range by means of smaller regression line elevations in *Cntnap2* KO rats compared with saline (most notably at 1.5 and 3 mg/kg, Figure 7G and Table 4, elevation: *p* = 0.0336). Slopes of the peak latency regression lines across the ASR dynamic range were not altered through R-Baclofen in comparison with saline within genotypes, neither in *Cntnap2* WT nor KO rats (Figures 7F–G and Table 4, slopes: WT *p* = 0.8086, KO *p* = 0.7055).

**Table 4:** Linear regression of ASR peak latencies in *Cntnap2* WT and KO rats after treatment with saline or R-Baclofen (0.75, 1.5, 3 mg/kg).

	RBac (mg/kg)	Saline	0.75	1.5	3
*Cntnap2 WT*	m	−5.900	−10.08	−10.92	−7.886
	c	306.5	327.6	327.7	334.0
	Sy.x	0.9501	4.142	6.339	8.221
RBac vs. Saline	m	N/A	0.2986^n.s.^	0.3836^n.s.^	0.7666^n.s.^
	c	N/A	0.0202*	0.0620^(*)^	0.0099**
*Cntnap2 KO*	m	0.05455	−0.004545	4.377	7.809
	c	295.8	294.4	271.8	263.6
	Sy.x	0.3860	12.25	7.991	4.045
RBac vs. Saline	m	N/A	0.9952^n.s.^	0.5246^n.s.^	0.1143^n.s.^
	c	N/A	0.8075^n.s.^	0.0375*	0.0272*

m: slope, c: Y-intercept, Sy.x: standard error of regression, RBac vs. Saline: within genotype comparison of regression lines after saline and R-Baclofen administration. *p* values, ^(*)^*p* < 0.1, *p < 0.05, **p < 0.01, n.s., not significant.

As shown in Figure 7 and Supplementary Figures 5, 6, R-Baclofen decreased ASR magnitudes in *Cntnap2* KO rats particularly at lower startle pulse intensities near ASR I-O threshold. To further analyze the effects of the three doses of R-Baclofen on peak latencies near ASR threshold, we performed linear regressions of the peak latencies at ASR I-O threshold across treatments (Figure 7H). Comparison of the two regression lines from *Cntnap2* WT and KO rats showed that the slopes were significantly different (Figure 7H, WT: *Y* = 5.893 * X + 306.6, Sy.*x* = 7.147; KO: Y = −8.876 * X + 295.3, Sy.*x* = 2.057, slopes *p* = 0.0116). In *Cntnap2* KO rats, the ASR peak latency at I-O threshold was negatively related to the R-Baclofen dose (i.e., the higher the dose, the shorter the latency, Figure 7H, slope *m* = −8.876 ms/increment, deviation from zero *p* = 0.0107, *F*_(1,2)_ = 91.68). No such relationship between latency and R-Baclofen dose was found in *Cntnap2* WT rats (Figure 7H, slope *m* = 5.893 ms/increment, deviation from zero *p* = 0.2088, *F*_(1,2)_ = 3.347). The differential effects of R-Baclofen on startle peak latencies at threshold in *Cntnap2* WT and KO rats became especially prominent at 3 mg/kg (Figure 7H, two-way RM ANOVA, treatment *p* = 0.8260, *F*_(2.290, 45.79)_ = 0.2271, genotype *p* = 0.0152, *F*_(1,20)_ = 7.050, treatment × genotype *p* = 0.1859, *F*_(3,60)_ = 1.657, Sidak’s multiple comparisons test, WT vs. KO: Saline *p* = 0.9991, 0.75 mg/kg *p* = 0.3525, 1.5 mg/kg *p* = 0.2288, 3 mg/kg *p* = 0.0021). This indicates that the cellular mechanisms or neural circuits controlling ASR peak latencies near ASR thresholds are affected differently by selective activation of GABA_B_ receptors through systemic administration of R-Baclofen in *Cntnap2* WT and KO rats.

### R-Baclofen Improves Sensorimotor Gating in *Cntnap2* KO Rats by Means of Increasing the Relative Amount and Relative Latencies of Startle in PPI Trials

**Figure 8:**
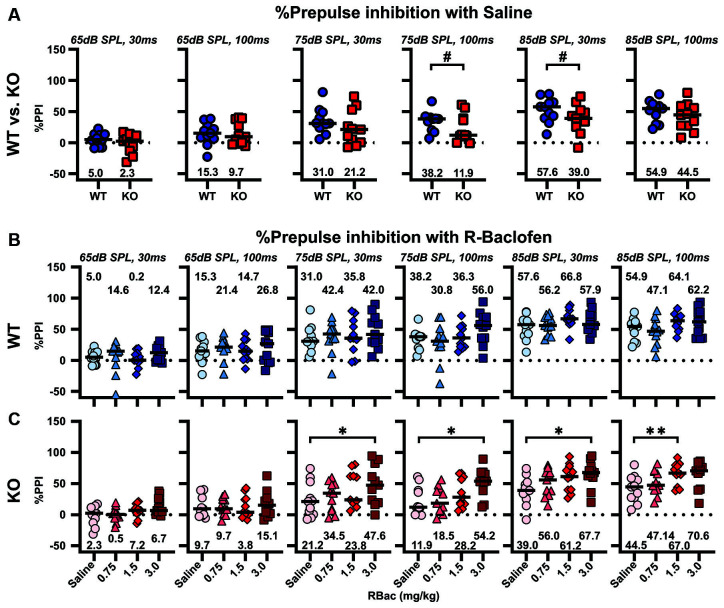
R-Baclofen increases the relative amount of PPI (%PPI) in *Cntnap2* KO rats. **(A–C)** %PPI was elicited by six different prepulse conditions with three stimulus levels at two different ISIs (from left to right: 65 dB SPL, 30 ms; 65 dB SPL, 100 ms; 75 dB SPL, 30 ms; 75 dB SPL, 100 ms; 85 dB SPL, 30 ms; 85 dB SPL, 100 ms). Scatter plots depict individual %PPI for each prepulse condition and black horizontal lines represent the median %PPI. **(A)** %PPI for each prepulse condition in *Cntnap2* WT (blue circles) and KO rats (red squares) after saline injection. *Cntnap2* KO rats had consistently, but statistically nonsignificant, lower %PPI than WT rats. Estimated *p* values of *p* = 0.0017 and *p* = 0.0163 indicate a significant PPI deficit in *Cntnap2* KO rats for prepulses with 75 dB SPL, 100 ms, and 85 dB SPL, 30 ms (for statistical comparisons and estimated *p* values through resampling see Table 5). **(B)** There were no significant differences in %PPI in *Cntnap2* WT rats between saline (circles), and 0.75 mg/kg (light blue triangles), 1.5 mg/kg (blue diamonds), and 3 mg/kg R-Baclofen (dark blue squares) for any of the six prepulse conditions (for statistical comparisons see Table 6). **(C)** In *Cntnap2* KO rats, %PPI was significantly increased through R-Baclofen (0.75 mg/kg: light red triangles, 1.5 mg/kg: red diamonds, 3 mg/kg R-Baclofen: dark red squares) compared with saline (circles), in particular with prepulses of 75 dB SPL, 30 ms; 75 dB SPL, 100 ms; 85 dB SPL, 30 ms (3 mg/kg); and 85 dB SPL, 100 ms (1.5 mg/kg). For statistical comparisons see Table 6. Dotted horizontal lines at 0%PPI represent no PPI of the startle. *p < 0.05; **p < 0.01; ^#^*p* < 0.05 estimated *p-*value through resampling.

The effect of R-Baclofen on sensorimotor gating in *Cntnap2* WT and KO rats was assessed using the PPI of the startle. The relative amount of PPI (%PPI) elicited by three prepulse stimulus levels (65, 75, and 85 dB SPL) at two different ISIs (30 and 100 ms) was first compared between *Cntnap2* WT and KO rats after injection of saline. *Cntnap2* KO rats had robust, but statistically nonsignificant, lower %PPI than WT rats for all prepulse conditions (Figure 8A and Table 5). Random permutation tests of %PPI for prepulses with 75 dB SPL, 100 ms, as well as 85 dB SPL, 30 ms between *Cntnap2* WT and KO rats gave estimated *p* values of *p* = 0.0017 and *p* = 0.0163 (40 repetitions of 10,000 random samples without replacement, see Table 5), indicating a significant PPI deficit in *Cntnap2* KO rats for these two prepulse types (Figure 8A and Table 5). In *Cntnap2* WT rats, R-Baclofen showed no significant effect on %PPI elicited by any of the six prepulse types (Figure 8B, statistical comparisons see Table 6). In contrast, KO rats showed a significant increase in %PPI in four of the six prepulse conditions through R-Baclofen (intensity, ISI: 75 dB SPL, 30 ms, 75 dB SPL, 100 ms, 85 dB SPL, 30 ms; 85 dB SPL, 100 ms, Figure 8C, for statistical comparisons see Table 6). In particular, %PPI in *Cntnap2* KO rats was increased with 1.5 mg/kg (prepulse 85 dB SPL, 100 ms) or 3 mg/kg R-Baclofen (prepulse 75 dB SPL, 30 ms, 75 dB SPL, 100 ms, 85 dB SPL, 30 ms; Figure 8C, for statistical comparisons see Table 6). Taken together, our results suggest that GABA_B_ receptor agonist R-Baclofen can improve deficient sensorimotor gating in *Cntnap2* KO rats by increasing the relative amount of PPI.

**Table 5:** Statistical comparison and estimated *p* values through resampling of %PPI elicited by six prepulse conditions in *Cntnap2* WT and KO rats after injection of saline.

Prepulse intensity, ISI	65 dB SPL, 30 ms	65 dB SPL, 100 ms	75 dB SPL, 30 ms	75 dB SPL, 100 ms	85 dB SPL, 30 ms	85 dB SPL, 100 ms
*Cntnap2 WT*	5.03 (−7.95–13.4)	15.3 (6.29–29.5)−	31.0 (24.2–48.8)	38.2 (19.6–40.4).	57.6 (39.4–64.9)	54.9 (29.5–62.4)
*Cntnap2 KO*	2.30 (−13.0–11.5)	9.67 (−1.99–38.0)	21.2 (0.308–53.0)	11.9 (9.82–38.9)	39.0 (25.4–52.2)	44.5 (27.4–59.0)
WT vs. KO						
Mann–Whitney test	0.4779^n.s.^	0.8977^n.s.^	0.1513^n.s.^	0.1513^n.s.^	0.1932^n.s.^	0.3316^n.s.^
WT vs. KO						
Estimated p value	0.4290^n.s.^	0.4599^n.s.^	0.1125^n.s.^	0.0017**	0.0163*	0.2869^n.s.^

Median (IQR); *p < 0.05, **p < 0.01, n.s.: not significant.

**Table 6:** Statistical comparison of %PPI within *Cntnap2* WT or KO rats after injection of saline, and 0.75, 1.5, and 3 g/kg R-Baclofen.

Prepulse intensity (dB SPL), ISI (ms)							
Genotype	Comparison	65 dB SPL, 30 ms	65 dB SPL, 100 ms	75 dB SPL, 30 ms	75 dB SPL, 100 ms	85 dB SPL, 30 ms	85 dB SPL, 100 ms
*Cntnap2 WT*	Friedman test	0.1828^n.s.^	0.6886^n.s.^	0.9209^n.s.^	0.2197^n.s.^	0.4088^n.s.^	0.5248^n.s.^
*Cntnap2 KO*	Friedman test	0.1039^n.s.^	0.5915^n.s.^	0.0394*	0.0017**	0.0456*	0.0252*
	Saline vs. 0.75 mg/kg	N/A	N/A	>0.9999^n.s.^	>0.9999^n.s.^	0.9653^n.s.^	0.1425^n.s.^
	Saline vs. 1.5 mg/kg	N/A	N/A	0.1425^n.s.^	0.4116^n.s.^	0.1425^n.s.^	0.0089**
	Saline vs. 3 mg/kg	N/A	N/A	0.0397*	0.0051**	0.0247*	0.1425^n.s.^

Post hoc test: Dunn’s multiple comparisons test; *p < 0.05, **p < 0.01, n.s.: not significant.

**Figure 9:**
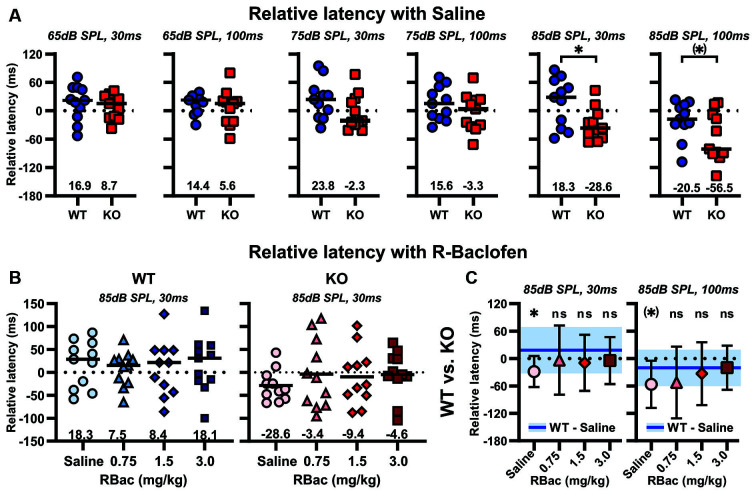
R-Baclofen increases the relative latencies of startle in PPI trials (ms) in *Cntnap2* KO rats compared with WT controls. **(A)** Relative latencies of startle in PPI trials for six different prepulse conditions with three stimulus levels at two different ISIs. Scatter plots depict individual relative latencies of startle in PPI trials for each prepulse condition and black horizontal lines represent the mean relative latency of startle. After saline injection, *Cntnap2* KO rats (red squares) had consistently shorter relative latencies of startle in PPI trials than WT rats (blue circles), with a significant difference for the prepulse condition 85 dB SPL, 30 ms and a tendency for the prepulse condition 85 dB SPL, 100 ms (two-sided student’s *t*-test, from left to right: 65 dB SPL, 30 ms; *p* = 0.5569; 65 dB SPL, 100 ms; *p* = 0.5043; 75 dB SPL, 30 ms; *p* = 0.1335; 75 dB SPL, 100 ms; *p* = 0.2566; 85 dB SPL, 30 ms; *p* = 0.0195; 85 dB SPL, 100 ms; *p* = 0.0819). **(B)** Scatter plots depicting individual (symbols) and mean relative latencies of startle in PPI trials (black horizontal lines) for prepulse condition 85 dB SPL, 30 ms in *Cntnap2* WT (**Left**, blue) and KO rats (**Right**, red) after injection of saline (circles), 0.75 mg/kg (triangles), 1.5 mg/kg (diamonds), and 3 mg/kg R-Baclofen (squares). R-Baclofen did not significantly increase relative latencies in *Cntnap2* WT (**Left**: WT, RM ANOVA, *p* = 0.9282, *F* = 0.1226). There was a slight, yet statistically not significant, increase in KO rats** (Right**: KO, RM ANOVA, *p* = 0.5611, *F* = 0.6374). **(C, Left)** Mean ± SD relative latency to prepulse condition 85 dB SPL, 30 ms in *Cntnap2* KO rats was significantly different from WT controls (after saline, blue line and area) with saline (two-sided student’s *t*-test, *p* = 0.0195), and similar to controls with 0.75 mg/kg (two-sided student’s *t*-test, *p* = 0.4381), 1.5 mg/kg (two-sided student’s *t*-test, *p* = 0.2627) and 3 mg/kg R-Baclofen (two-sided student’s *t*-test, *p* = 0.3069).** (C, Right)** Mean ± SD relative latency to prepulse condition 85 dB SPL, 100 ms in *Cntnap2* KO rats was by tendency different from WT controls (after saline, blue line and area) with saline (two-sided student’s *t*-test, *p* = 0.0819), and similar to controls with 0.75 mg/kg (Welch’s *t*-test, *p* = 0.2479), 1.5 mg/kg (two-sided student’s *t*-test, *p* = 0.6028) and 3 mg/kg R-Baclofen (two-sided student’s *t*-test, *p* = 0.9833). Dotted horizontal lines at 0 Relative Latency (ms) represent similar latency to the maximum startle response in trials with vs. without a prepulse. ^(*)^*p* < 0.1, *p < 0.05; n.s.: not significant.

To analyze the influence of R-Baclofen on temporal properties of sensorimotor gating, we compared the change in latency to the maximum startle response in trials with and without a prepulse between *Cntnap2* WT and KO rats (Figure 9). After injection of saline, *Cntnap2* KO rats showed generally shorter relative latencies than WT rats. The difference was significant for relative latencies to the prepulse type with 85 dB SPL, 30 ms (Figure 9A, two-sided student’s *t*-test *p* = 0.0195). The shorter relative latencies in trials that included a prepulse indicated impaired temporal characteristics of sensorimotor gating in *Cntnap2* KO rats compared to WT rats. Within genotype, comparisons showed that R-Baclofen did not significantly increase the relative latencies in either *Cntnap2* WT or KO rats for any of the six prepulse types, even though there appeared to be a slight increase in relative latency for some prepulse conditions in *Cntnap2* KO rats (shown for prepulse condition 85 dB SPL, 30 ms in Figure 9B, Left: WT, RM ANOVA, *p* = 0.9282, *F* = 0.1226; Right: KO, RM ANOVA, *p* = 0.5611, *F* = 0.6374; for statistical results of all six prepulse conditions see Supplementary Figure 8). Therefore, we aimed to analyze if subtle changes in relative latency through R-Baclofen had the potential to increase latencies in *Cntnap2* KO rats to WT control levels after saline injection. Indeed, all three doses of R-Baclofen increased the relative latency in *Cntnap2* KO rats to levels similar to WT controls for prepulse type 85 dB SPL, 30 ms (Figure 9C, two-sided student’s *t*-test, WT—Saline vs. KO–0.75 mg/kg: *p* = 0.4381, WT—Saline vs. KO–1.5 mg/kg: *p* = 0.2627, WT—Saline vs. KO–3 mg/kg: *p* = 0.3069). This indicates that GABA_B_ receptor agonist R-Baclofen can improve deficient sensorimotor gating in *Cntnap2* KO rats by subtle increases of the relative latency of startle in PPI trials with a minimal dose of 0.75 mg/kg R-Baclofen.

## Discussion

The present study sought to investigate whether selective activation of GABA_B_ receptors can remediate ASD-related altered sensory processing reliant on auditory brainstem function. We, therefore, compared behavioral read-outs of brainstem auditory signaling from rats with the homozygous knockout of *Cntnap2* to their WT littermates, with and without administration of R-Baclofen. Homozygous loss-of-function of *Cntnap2* leads to characteristic changes in brainstem-mediated auditory processing and behavior (Scott et al., 2018, 2020). Here, we demonstrate that these functional changes are accompanied by increased levels of excitatory and inhibitory neurotransmitters in the startle-mediating PnC and that they can largely be remediated by selective activation of GABA_B_ receptors through R-Baclofen. In the present study, R-Baclofen: (1) improved deficient sensory filtering by enhancing short-term habituation; (2) suppressed exaggerated responses to moderately loud startling sounds; (3) rectified dynamic range response characteristics including ASR threshold, half-maximal response, and saturation; (4) improved sensorimotor gating by means of the relative amount of PPI and latency of startle in PPI trials; (5) but did not improve startle sensitization and peak response latency at ASR threshold in *Cntnap2* KO rats. Therefore, our results provide evidence that GABA_B_ receptor agonists may be useful for pharmacologically targeting multiple aspects of sensory processing disruptions in ASD.

### E/I Imbalance in *Cntnap2* KO Rats

Perturbed balance in neuronal excitation and inhibition is commonly assumed a possible final shared mechanism in autism (for review, see Rubenstein and Merzenich, 2003) that might underlie altered auditory processing in ASD (for review, see Sinclair et al., 2017b). *Cntnap2* is suggested to be involved in the regulation of neuronal circuit E/I balance, evidenced by decreased dendritic arborization and spine development after *Cntnap2* knockdown in cortical neurons (Anderson et al., 2012), and by increased excitatory synaptic input (Scott et al., 2017) and disrupted maturation of GABAergic inhibitory transmission in the cortex of *Cntnap2* KO mice (Bridi et al., 2017). Given the expression of *Cntnap2* along the ascending auditory and startle-mediating pathways (Figure 1)—including auditory nerve, dorsal, ventral, and granular layers of the cochlear nucleus (CN), SOC, dorsal nucleus of the lateral lemniscus, inferior colliculus, medial geniculate body, CRN, PnC, and pedunculopontine tegmental nucleus (PPT; Gordon et al., 2016; Scott et al., 2018)—it is plausible to assume that an irregular E/I balance in the auditory brainstem from *Cntnap2* KO rats is underlying the ASD-like altered implicit auditory-evoked behaviors observed in the present study (Figures 3, 8A, 9A). Indeed, quantification of amino acid levels through MALDI MS imaging demonstrated an increase in glutamine, glutamate, and GABA in the PnC from *Cntnap2* KO rats (Figure 4). Herein, GABA appeared to be disproportionally elevated, evidenced by lower Glu/GABA, increased GABA/Glu, but similar Glu/Gln ratio compared with WT controls. Due to the limited spatial resolution of MALDI imaging (80 μm), we can neither draw conclusions about the (sub-) cellular localization (extra- or intracellular, neuronal or glial, vesicular or cytoplasmic) of the detected amino acids, nor about the availability of the neurotransmitters for synaptic signaling (Waagepetersen et al., 2001; for reviews see Choudhury et al., 2011; Coghlan et al., 2012). Astrocytic-derived glutamine is the precursor of both glutamate and GABA. Normally, more glutamine is transferred from astrocytes to glutamatergic neurons, since GABAergic neurons have a greater capability of re-utilizing their neurotransmitter by re-uptake (for review, see Walls et al., 2015). The perturbed relations between Gln, Glu, and GABA in the PnC from *Cntnap2* KO rats indicate a dysregulation in the glutamine-glutamate/GABA re-uptake and/or synthetization cycle that might result in disturbance of the functional E/I homeostasis and underlie ASD pathogenesis (Van Elst et al., 2014). These alterations could be the result of a compensatory upregulation of neurotransmitter levels in response to decreased postsynaptic receptor availability, as has been observed in form of decreased glutamate receptor expression in the PFC from *Cntnap2* KO mice (Kim et al., 2019), impaired glutamate receptor trafficking to the cell surface of hippocampal *Cntnap2* KO neurons (Varea et al., 2015), and reduced GABA receptor subunit expression in autistic human brain samples (Fatemi et al., 2009; Blatt and Fatemi, 2011). A compensatory upregulation of GAD67, one of the isoforms of the key synthesizing enzymes for GABA, has been observed in ASD brains, possibly to provide increased GABAergic feed-forward inhibition to compensate for the loss of cerebellar neurons (Yip et al., 2008). Interestingly, reduced numbers of cortical GABAergic interneurons have also been observed in *Cntnap2* KO mice (Peñagarikano et al., 2011) and reduced numbers of neurons in the auditory brainstem of humans with ASD (Kulesza et al., 2011). It remains to be elucidated whether *Cntnap2* KO rats have reduced numbers of neurons in the PnC and whether the elevated GABA level observed in our study is correlated with (insufficient) compensatory upregulation (Antoine et al., 2019) of GABAergic feedforward inhibition from the PPT to the PnC (Yeomans et al., 2010; Fulcher et al., 2020). It should be noted that abnormalities in glutamine, glutamate, and GABA levels appear to be highly age-, species-, strain-, and brain region/circuit-specific (Horder et al., 2013, 2018a,b; Van Elst et al., 2014). Interestingly, GABA, Glu, and Gln levels were not altered in the SOC within the auditory brainstem of *Cntnap2* KO rats (Supplementary Figure 4). This indicates that *Cntnap2* might not generally interact with the Glu-Gln/GABA system throughout the brain. It rather suggests that Glu-Gln/GABA system dysregulation might be a secondary effect of functional *Cntnap2* deletion that is confined to certain brain regions or neural circuits. Analogous to our findings in the PnC from *Cntnap2* KO rats, BTBR T+tf/J mice show increases in all three amino acids particularly in the striatum, but not in the PFC (Horder et al., 2018b). Several other rodent models of ASD presented with other distinct Glu, Gln, and GABA concentration profiles, three of them recapitulating the reduction in striatal Glu from the adult autistic human cohort (Horder et al., 2018b). Higher absolute concentrations of GABA and glutamate, as well as lower Glu/GABA ratios, on the other hand, have been described in blood plasma from pediatric and adolescent autistic patients (El-Ansary and Al-Ayadhi, 2014; Al-Otaish et al., 2018). Increased combined Gln and Glu signals in the anterior cingulate from children and adolescents with ASD have been interpreted as an indicator of neuronal overexcitation (Bejjani et al., 2012; Van Elst et al., 2014), and increased GABA—a product of glutamate metabolism—as a consequence of significantly elevated glutamate and/or decreased breakdown of GABA into glutamate (El-Ansary and Al-Ayadhi, 2014; for review, see Walls et al., 2015; Zheng et al., 2019). Given that baseline ASRs rely on the glutamatergic excitation of PnC giant neurons (Ebert and Koch, 1992), and GABA receptors on PnC giant neurons mediate a substantial part of PPI (Yeomans et al., 2010), the dysregulation of the Glu-Gln/GABA system likely perturbs acoustic startle circuitry and behavior in *Cntnap2* KO rats. Even though future immunohistochemical and electrophysiological studies are needed to investigate the anatomical distribution and functional correlation of amino acid levels in the startle-mediating pathway in closer detail, our results strongly indicate that altered implicit auditory-evoked behaviors commonly observed in ASD (Chamberlain et al., 2013; Kohl et al., 2014; Takahashi et al., 2016) might result from disturbed E/I balance within the neuronal startle circuit.

### R-Baclofen Mechanism of Action

At this point, we can only speculate how *R*-Baclofen treatment improves the behavioral read-outs of sensory processing in *Cntnap2* KO rats. The efficacy of R-Baclofen could result from its ability to dampen hyperexcitability *via* pre- and postsynaptic mechanisms. Baclofen stimulates metabotropic GABA_B_ receptors which function as presynaptic auto- or heteroreceptors to inhibit the vesicular release of GABA or glutamate, respectively (Waldmeier et al., 2008; Delaney et al., 2018). Postsynaptically, R-Baclofen activates inward-rectifying potassium channels that cause neuronal hyperpolarization. Together, these mechanisms serve to tonically hyperpolarize neurons, decrease resting membrane potential, and reduce cell firing (Gandal et al., 2012; for review, see Wu and Sun, 2015). R-Baclofen may be beneficial in *Cntnap2* KO animals by counteracting the reported reduction in the number of GABAergic interneurons and asynchronous neuronal firing (Peñagarikano et al., 2011; Vogt et al., 2017), decreased GABAergic phasic and tonic inhibition (Bridi et al., 2017), increased neurotransmitter release and increased postsynaptic excitatory responses in *Cntnap2* KO animals (Scott et al., 2017), and the dysregulated glutamine-glutamate/GABA cycle indicated by the lacking rebalance of Glu/GABA ratios in the present study.

In the auditory system, Baclofen has been shown to have large effects on overall excitability (Szczepaniak and Møller, 1995), including the suppression of sound-evoked activity and/or hyperexcitability in the CN (Martin, 1982; Caspary et al., 1984), inferior colliculus (Szczepaniak and Møller, 1996; Sun et al., 2006) and auditory cortex (Lu et al., 2011). In a genetic mouse model of E/I dysfunction, Baclofen dose-dependently normalized auditory-evoked potentials, elevated ASRs, and deficient PPI of ASRs. This was linked to the improvement of several elements of E/I homeostasis such as circuit excitability, neural synchrony, and signal-to-noise ratio (Gandal et al., 2012).

ASR amplitudes are the sum of habituation and the parallel independent process of sensitization, with habituation being the decrease and sensitization the initial increase in magnitude to a series of sound pulses (Payne and Anderson, 1967; Groves and Thompson, 1970; Geyer and Braff, 1982; Pilz and Schnitzler, 1996; Rankin et al., 2009). Impaired habituation and increased sensitization apparent in our study in *Cntnap2* KO rats (Figures 3B,C) are also associated with ASD in humans (Perry et al., 2007; Chamberlain et al., 2013; Madsen et al., 2014). Short-term habituation relies on synaptic depression at the axon terminals of the CRN sensory afferents in the PnC (Figure 1), likely mediated by activation of voltage- and calcium-dependent potassium channels (Ebert and Koch, 1992; Weber et al., 2002; Simons-Weidenmaier et al., 2006; Zaman et al., 2017). Lack of *Cntnap2* in KO rats might interfere directly with startle habituation through its function in clustering of voltage-gated potassium channels (Poliak et al., 2003; Dawes et al., 2018; Scott et al., 2018). At an auditory glutamatergic synapse featuring strong synaptic depression, Baclofen modulated transmitter release in an activity-dependent manner (Brenowitz et al., 1998) which might explain the improvement of short-term habituation in *Cntnap2* WT and KO rats with R-Baclofen (Figures 5A–C). Sensitization, on the other hand, is caused by extrinsic modulation of the startle pathway (Figure 1) by structures including the periaqueductal gray, the amygdala, and the bed nucleus of the stria terminalis (Leaton and Supple, 1986; Fendt et al., 1994a, b; Davis et al., 1997)—structures that all express *Cntnap2* (Alarcón et al., 2008; Gordon et al., 2016). The ineffectiveness of R-Baclofen to suppress increased ASR sensitization in *Cntnap2* KO rats or sensitization in WT controls (Figure 5D) might be due to the fact that the modulatory input from these structures altering sensitization includes several neurotransmitters other than GABA or Glu, such as noradrenaline (Fendt et al., 1994a), substance P (Krase et al., 1994), glycine (Plappert et al., 2001), or dopamine (Halberstadt and Geyer, 2009). In support of this notion, Baclofen was also unable to reverse dopamine receptor agonist apomorphine-induced disruptions in sensorimotor gating, while it did reverse NMDA receptor antagonist effects (Bortolato et al., 2004).

Baclofen and its formulations R- and S-Baclofen are well known to suppress ASRs in controls and in animals with proposed E/I dysfunction, either genetically or pharmacologically induced (e.g., Bortolato et al., 2004; Lu et al., 2011; Gandal et al., 2012). In our hands, R-Baclofen was more potent in *Cntnap2* WT rats (effective dose of 0.75 mg/kg) than in *Cntnap2* KO rats (effective dose of 1.5 mg/kg, Figure 6) and more effective in female than in male KO rats (Supplementary Figure 5). This sex-dependent effect of R-Baclofen on the ASR I-O function is probably due to the fact that *Cntnap2* KO males had higher ASR magnitudes than females to begin with (i.e., without R-Baclofen, Figures 3D,E). Sex effects on ASR I-O function in untreated rats from the *Cntnap2* model have been described before (Scott et al., 2018). In humans, the male prevalence of ASD symptoms has been attributed to sex-differential factors such as reduced susceptibility in females, or lower mutational burden threshold in males. In this regard, mutations affecting GABA signaling appear to be particularly pervasive in males (for reviews, see Werling and Geschwind, 2013; Rylaarsdam and Guemez-Gamboa, 2019), and *Cntnap2* mutations affect functional responses of cortical circuitry more strongly in male than in female mice (Townsend and Smith, 2017). Interestingly, the *Cntnap2* gene is differentially expressed in sexually dimorphic song nuclei essential for vocal learning in songbirds (Panaitof et al., 2010) in accordance with the sexual dimorphism of neural circuitry in vocal control areas (Nottebohm and Arnold, 1976); and genetic variants in the *CNTNAP2* gene are associated with gender differences among dyslexic children (Gu et al., 2018). Exploring in more detail the neurobiological basis of sex-dependent differences in startle responses and efficacy of R-Baclofen found in *Cntnap2* KO rats should be considered in future studies.

In addition to differences in the effective dose, R-Baclofen suppressed ASR magnitudes across a wide range of startle pulse intensities in *Cntnap2* WT rats, whereas in KO rats the maximum ASR capacity was unaltered (Figure 6). A similar phenomenon has been described in rats after treatment with S-Baclofen to suppress salicylate-induced enhancement of ASRs (Lu et al., 2011). The robustly increased ASRs to high sound intensities in *Cntnap2* KO rats might be due to increased excitatory input from the CN to the PnC (Figure 1). Behavioral studies showed that electrolytic lesions of the CN reduced ASRs particularly to loud sound intensities of 110 and 115 dB SPL (Meloni and Davis, 1998). In contrast, chemical lesions of CRNs or the PnC blocked ASRs at all intensities (Lee et al., 1996). Interestingly, Flores et al. (2015) identified an alternative pathway from the cochlea to the CN for the detection of loud, potentially tissue-damaging, auditory stimuli. One might speculate if this form of sensation (termed “auditory nociception”) is increased in *Cntnap2* KO rats and contributes to their exaggerated ASRs (Figure 3) as well as greater active sound avoidance (Scott et al., 2020). “Auditory nociception” would have similarities to C-fiber nociception (Flores et al., 2015) which is indeed enhanced in *Cntnap2* KO animals (Dawes et al., 2018). Taken together, the dramatically reduced ASRs including maximum capacity in *Cntnap2* WT rats by R-Baclofen (Figure 6) might be predominantly due to reduced excitability in CRNs and/or the PnC. In contrast, in *Cntnap2* KO rats, the R-Baclofen-induced suppression of exaggerated responses to moderate startling sounds might be the behavioral outcome of an interaction between reduction in CRNs and/or PnC hyperexcitability, and unproportionally high excitatory input from the CN to PnC.

The decrease of ASRs to moderate startle pulse intensities through R-Baclofen in *Cntnap2* KO rats was accompanied by the normalization of their ASR thresholds to control levels, indicated by an increase of the minimum sound intensity required to elicit a response (from about 83–89 dB SPL at the 25% response magnitude, Figure 7, Supplementary Table 2). The high acoustic input required to reach the ASR threshold and elicit a motor response is likely determined by a high firing threshold in the CRNs. In contrast, electrophysiological data have shown that PnC neurons, that receive rapid input from the CRNs, have a relatively low firing threshold (Wagner and Mack, 1998; Brosda et al., 2011). Given the expression of *Cntnap2* in CRNs from WT rats (Scott et al., 2018), its lack in *Cntnap2* KO rats may result in neuronal hyperexcitability in the CRNs, leading to lower ASR thresholds (Figure 3H). CRN neurons receive inhibitory GABAergic input that modulates their neuronal responses and consequently the ASR output (for review, see Osen et al., 1991; Gómez-Nieto et al., 2008). Therefore, R-Baclofen might attenuate intrinsic excitability and increase firing thresholds of CRNs, and thereby normalize ASR thresholds in *Cntnap2* KO rats. Alternatively, R-Baclofen might take effect by blocking the glutamate release from the auditory nerve fibers (Martin, 1982) synapsing onto CRNs (Gómez-Nieto et al., 2014).

The normalization of the ASR threshold in *Cntnap2* KO rats through R-Baclofen was correlated with a parallel rightward shift of the I-O function, determined by an increase in the half-maximal response (ES50) and ASR saturation (90% response magnitude, Figure 7, Table 3). This means that—while the extent of the I-O dynamic range remained similar—the I-O dynamic range was shifted to higher startle pulse intensities. Conversely, this indicates that the acoustic stimulus potency was decreased by R-Baclofen. In the dynamic range of the I-O function, a small stimulus change can produce a large response change (Stoddart et al., 2008) and the slope is an important aspect of the ASR I-O function as it directly reflects the sensorimotor integration process (Hince and Martin-Iverson, 2005). R-Baclofen did not induce a change in ASR I-O slope within the genotype (Supplementary Figure 7 and Supplementary Table 3). Therefore, it can be assumed that in *Cntnap2* KO rats the ASR efficiency, i.e., the transduction of sensory information into motor output, remained at a similar rate (Figure 2). This speaks against a generalized increase in inhibition of the ASR system through R-Baclofen, as this would also predict a change in slope (Hince and Martin-Iverson, 2005). Interestingly, Martin-Iverson and Stevenson (2005) found a change in ASR I-O slope through emotional modulatory input such as fear, modified by dopaminergic signaling (Figure 1). It should be pointed out that R-Baclofen normalized the ASR I-O fitted curves from *Cntnap2* KO rats to control levels, despite the unaltered slope in the within genotype comparisons (Figures 7A–C, Supplementary Figure 7).

ASR magnitude and latency are in general negatively correlated (i.e., the higher the magnitude, the shorter the latency; Hoffman and Searle, 1968). In a unique approach, we analyzed ASR peak latencies from individual animals relative to their dynamic range characteristics (i.e., threshold, ES50, saturation, Figures 7F–H). This allowed us to investigate the processing speed between sensory (acoustic) input and maximum ASR motor output without the confounding effect of genotype-related differences in startle magnitudes. Peak latencies were significantly shorter in *Cntnap2* KO than in WT rats, specifically at the ASR threshold. Surprisingly, R-Baclofen led to even shorter, rather than longer, ASR peak latencies in *Cntnap2* KO rats. As outlined above, motor responses to low, near-threshold, acoustic inputs are likely gated by CRN activity (Wagner and Mack, 1998; Brosda et al., 2011). It might be possible that the shift in threshold to higher sound intensities in *Cntnap2* KO rats with R-Baclofen goes along with more synchronous short-latency inputs to the PnC, thereby speeding up temporal processing (Gandal et al., 2012; Harris and Dubno, 2017). In contrast to GABA_B_ receptor activation through R-Baclofen, pharmacological modulation of other neurotransmitter receptors targeting ASR sensitization might have shown normalizing effects on ASR latency, since the course of response latency is dominated by ASR sensitization (Pilz and Schnitzler, 1996).

In addition to increased acoustic reactivity, *Cntnap2* KO rats consistently presented with disrupted sensorimotor gating in two of our previous studies, despite differences in the acoustic prepulse conditions (Scott et al., 2020) or breeding scheme (Scott et al., 2018). In line with these previous results, *Cntnap2* KO rats in the present study also displayed robustly lower PPI of ASRs than WT controls (Figure 8A). These differences were statistically significant for two prepulse conditions (75 dB SPL, 100 ms and 85 dB SPL, 30 ms ISI) with a random permutation test for small sample sizes. R-Baclofen improved sensorimotor gating in *Cntnap2* KO rats as shown by a dose-dependent increase in PPI for four prepulse conditions (75 dB SPL, 30 ms; 75 dB SPL, 100 ms; 85 dB SPL, 30 ms; 85 dB SPL, 100 ms, Figure 8C). Likewise, enhancing GABAergic inhibition through Baclofen previously rescued PPI disrupted by pharmacological NMDA receptor blockade (Bortolato et al., 2004; Arai et al., 2008; Fejgin et al., 2009) or hypofunction (Gandal et al., 2012). In control animals, Baclofen *per se* produced no significant changes in PPI at any given dose in these previous studies (Bortolato et al., 2004), similar to *Cntnap2* WT rats in our study (Figure 8B). This was due to the uniform suppression of response magnitudes in trials with and without a prepulse (Supplementary Figure 9A). In contrast, in *Cntnap2* KO rats the response magnitudes to the prepulse + startle pulse condition were suppressed more strongly by R-Baclofen than the ones to the startle pulse alone condition (Supplementary Figure 9B). Previous studies have demonstrated the involvement of GABA_B_ receptors in prepulse processing and sensorimotor gating (Koch et al., 2000; Takahashi et al., 2007; Yeomans et al., 2010). R-Baclofen might improve the behavioral salience of weak acoustic prepulses through increased feedforward inhibition onto the PnC (Carlson and Willott, 1996; Price et al., 2008; Antoine et al., 2019) achieved by decreased spontaneous firing (“neuronal noise”) and improved neural synchrony in response to the prepulse (Gandal et al., 2012) within the PPI circuitry (Figure 1). It is unlikely that the improved sensorimotor gating was due to changes in detectability of the prepulse in the auditory periphery (i.e., hearing thresholds) since Baclofen does not affect the sound-evoked cochlear output and summed auditory nerve potentials (Martin, 1982). In addition, the prepulse elicited response (at 100 ms, Supplementary Figure 10) was not increased with R-Baclofen, which is different from a pharmacologically induced rodent model of schizophrenia-like sensorimotor gating deficits (Yee et al., 2004).

In line with our previous results (Scott et al., 2018), *Cntnap2* KO rats did not only show disrupted PPI in terms of amplitudes but also a lack of the typical increase in startle latency in PPI trials (Figure 9A; Ison et al., 1973; Hoffman and Ison, 1980). However, in contrast to ASR peak latencies (at the threshold, Figures 7F–H), R-Baclofen prolonged and normalized the startle latency in PPI trials from *Cntnap2* KO rats (Figure 9C). This might underscore that the changes in neuronal transmission rectifying not only PPI amplitudes but also latencies mainly lie within the circuit branch processing prepulse information and take effect downstream of the CRN (i.e., GABAergic PPT projections onto PnC).

### Model Validity and Clinical Implications

Even though we cannot fully exclude dose-dependent myorelaxant properties of R-Baclofen (Davidoff, 1985; Nevins et al., 1993), it is reasonable to assume that the changes we observed in *Cntnap2* KO rats were mostly due to the brainstem processing involved in ASR generation. This is because the maximum ASR as a putative index for motor capacity (Hince and Martin-Iverson, 2005) was not altered in *Cntnap2* KO rats even with 3 mg/kg R-Baclofen, and ASR peak latencies at the threshold were shortened, not prolonged. Importantly, intrathecal administration of Baclofen reversed enhanced ASRs and restored reduced PPI of the blink reflex in patients with spinal cord injury, strongly suggesting a muscle tone regulating effect of Baclofen at the brainstem level (Kumru et al., 2009; Kumru and Kofler, 2012). Future studies should address in more detail the sites and mechanisms of R-Baclofen action. The most promising target of R-Baclofen action is the PnC as it is the sensorimotor interface of the startle circuit (Figure 1), where the transition of sensory input into the motor output can be directly influenced (for review, see Koch, 1999). Using cannulated microelectrodes, R-Baclofen infusions into the PnC and simultaneous electrophysiological recordings in behaving *Cntnap2* WT and KO rats would allow to assess changes in startle responses correlated to changes in PnC neuronal activity without possible systemic effects of R-Baclofen. Suppression of the speculated PnC hyperexcitability in *Cntnap2* KO rats through local application of R-Baclofen might attenuate their exaggerated startle responses, in particular to moderate startling sounds. Furthermore, microinfusions of R-Baclofen to the cochlear round window membrane might be a useful tool to dissect the contribution of the sensory (as opposed to motor) branch in the ASR pathway to effects observed in our study. The round window membrane delivery approach of R-Baclofen to the inner ear might reduce glutamate release from the auditory nerve fibers synapsing onto CRNs (Gómez-Nieto et al., 2014), resulting in less sound-evoked PnC activity, and possibly a shift in ASR thresholds as well as reduced startle response magnitudes. Lastly, R-Baclofen-induced alterations in modulatory input to the PnC might be identified through local application to the PPT. Simultaneous electrophysiological recordings of sound-evoked activity in PnC neurons to a prepulse+startle pulse sound paradigm would help scrutinize R-Baclofen-induced changes in GABAergic feedforward inhibition from the PPT to the PnC that might underlie altered PPI of startle in *Cntnap2* KO rats in the present study. On a cellular level, R-Baclofen actions on excitatory and inhibitory transmission (mediated by presynaptic GABA_B_ heteroreceptors or autoreceptors, respectively) could be addressed by examining its effects on excitatory (glutamatergic) and inhibitory (GABAergic) postsynaptic currents using whole-cell voltage clamp recordings in PnC giant neurons from *Cntnap2* WT and KO rats.

Rats with homozygous, and to a lesser extent heterozygous, functional deletion of the *Cntnap2* gene display behavioral alterations that parallel core symptoms of ASD, including deficits in sociability, repetitive stereotypy, and sensory abnormalities (Scott et al., 2018, 2020). Therefore, the *Cntnap2* rat model for autism does not only have a high construct but also face validity. This is particularly important considering that ASD diagnosis and consequently validation of treatments rely on evaluating behavioral traits both in clinical testing and in preclinical models that seek to recapitulate those behavioral traits from humans (for reviews, see Servadio et al., 2015; Kazdoba et al., 2016; Möhrle et al., 2020; Scott et al., 2021). One limitation of our study might be that single gene mutations such as *Cntnap2* account for no more than 1% of ASD cases (for review, see Yoo, 2015). However, the majority of ASD susceptibility genes seem to converge in shared or interacting biological pathways that are typically involved in synapse formation and function, transcriptional control, and chromatin-remodeling (De Rubeis et al., 2014; Iossifov et al., 2014; Pinto et al., 2014). Therefore, monogenic rodent models including *Cntnap2* are useful tools in the search of standardized objective biomarkers for the neurological basis, and the utility of diagnosis and treatment of ASD.

Exaggerated acoustic reactivity and impaired sensorimotor gating have been described in individuals with autism (Perry et al., 2007; Chamberlain et al., 2013; Kohl et al., 2014; Takahashi et al., 2016) along with other sensory alterations affecting the auditory, visual, touch, smell/taste and pain domain. Exploring the usefulness of therapeutic approaches to rectify sensory alterations might be of particular importance considering that atypical low-level sensory processing might exacerbate or interact with other, higher-level, symptoms in individuals with ASD (O’Neill and Jones, 1997; Leekam et al., 2007). For example, regarding the auditory system, timing deficits within the brainstem negatively impact rapid acoustic processing, predictive of a higher risk for developing speech processing issues and language disorders (Benasich et al., 2002; Wible et al., 2004; Abrams et al., 2006), representing core symptoms of ASD (for review, see Alarcón et al., 2008; Mody and Belliveau, 2013; Rodenas-Cuadrado et al., 2016). Interestingly, rodent models with mutations in *Cntnap2* parallel slowed neurotransmission along the ascending auditory brainstem reported in ASD (Rosenhall et al., 2003; Kwon et al., 2007; Miron et al., 2016; Scott et al., 2018), and deficient language-relevant rapid auditory processing seen in infants carrying variants of *Cntnap2* (Truong et al., 2015; Riva et al., 2018). Targeting E/I balance to modulate more spectrotemporally complex auditory processes such as brainstem representation and higher-level perception of speech-like sounds in *Cntnap2* KO rats is an exciting consideration for future studies.

## Conclusion

In conclusion, this study demonstrated a relationship between *Cntnap2* gene deletion, disrupted excitatory/inhibitory homeostasis, auditory brainstem-mediated sensory processing, and symptoms of ASD. Increasing GABAergic signaling *via* the GABA_B_ receptor agonist R-Baclofen improved many aspects of acoustic reactivity, sensory filtering, and sensorimotor gating in *Cntnap2* KO rats. These findings encourage further efforts to establish translatable paradigms based on auditory-evoked behaviors for preclinical and clinical therapeutic screening for neurodevelopmental disorders. Our results support the hypothesis that enhancing inhibitory transmission improves ASD relevant deficits and that GABA_B_ receptors are a promising therapeutic target for restoring neural circuit and behavioral abnormalities in disorders characterized by E/I imbalance.

## Data Availability Statement

The raw data supporting the conclusions of this article will be made available by the authors, without undue reservation.

## Ethics Statement

The animal study was reviewed and approved by the University of Western Ontario Animal Care Committee, and all procedures were in accordance with the guidelines established by the Canadian Council on Animal Care.

## Author Contributions

DM, SW, and SS: participated in research design. DM and WW: conducted experiments. DM: performed data analysis. DM, WW, SW, and SS: wrote or contributed to the writing of the manuscript. All authors contributed to the article and approved the submitted version.

## Conflict of Interest

The authors declare that the research was conducted in the absence of any commercial or financial relationships that could be construed as a potential conflict of interest.

## Publisher’s Note

All claims expressed in this article are solely those of the authors and do not necessarily represent those of their affiliated organizations, or those of the publisher, the editors and the reviewers. Any product that may be evaluated in this article, or claim that may be made by its manufacturer, is not guaranteed or endorsed by the publisher.
