# Supplementary material for: GABAB Receptor Agonist R-Baclofen Reverses Altered Auditory Reactivity and Filtering in the Cntnap2 Knock-Out Rat
Source: Front Integr Neurosci. 2021 Aug 20;15:710593. doi: 10.3389/fnint.2021.710593 (PMC8417788; doi:10.3389/fnint.2021.710593)
Supplement: Supplementary file 1 [file Data_Sheet_1.pdf]

Supplementary Material

1 Supplementary Figures

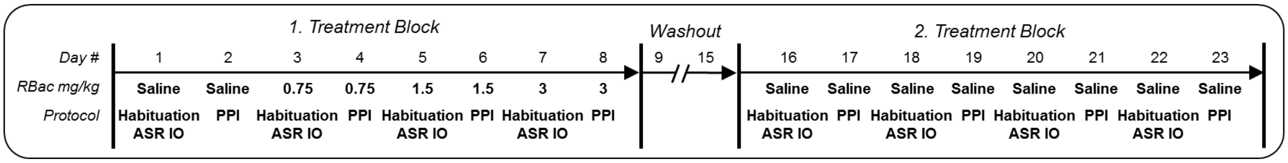

Supplementary Figure 1. Experimental design to investigate the effects of R-Baclofen on implicit auditory-evoked behaviors. In the first treatment block, each of the four different treatments was applied on two consecutive days, beginning with the vehicle treatment (saline), and then R-Baclofen in increasing doses (0.75, 1.5, 3 mg/kg body weight *i.p.)*. On the first of the two treatment days, the behavioral procedures for sensory filtering (habituation) and acoustic reactivity (acoustic startle response input-output, ASR IO) were performed, and on the second day for sensorimotor gating (prepulse inhibition, PPI). After the first treatment block, the animals were given a washout break of one week. In the second treatment block, the behavioral procedures were repeated with the same sequence after saline injections to control for effects of repeated testing.

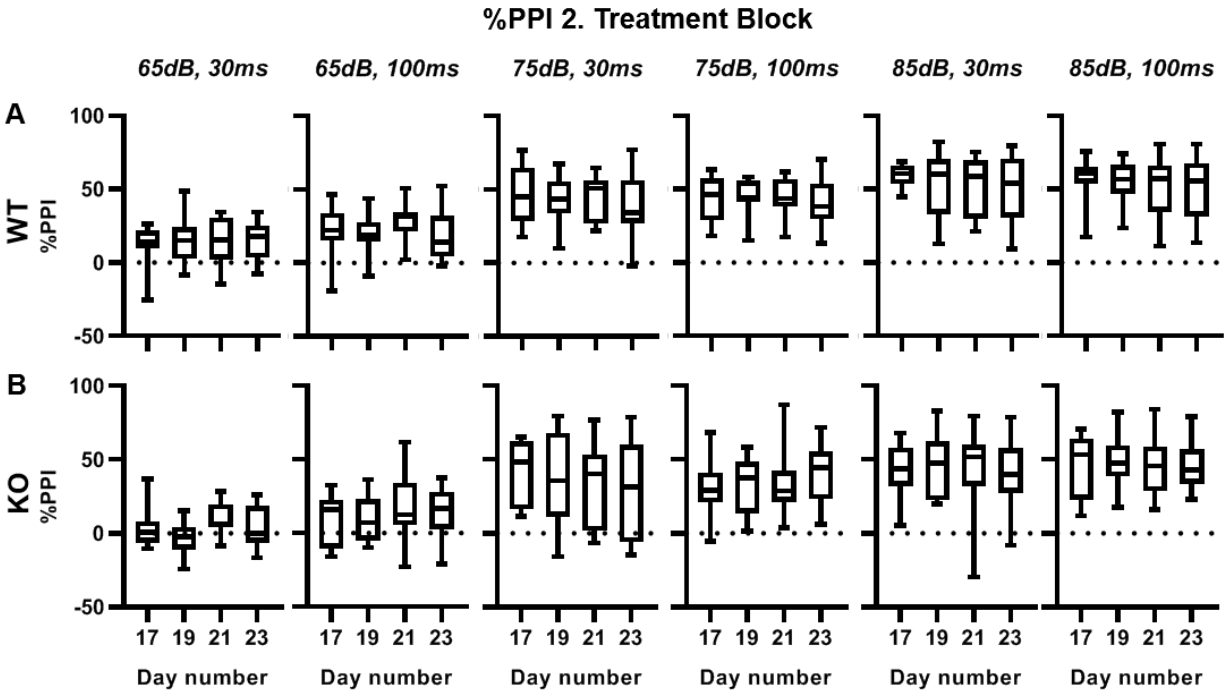

Supplementary Figure 2. Repeated testing within the 2. Treatment Block did not significantly alter relative amount of PPI in *Cntnap2* WT or KO rats. The relative amount of PPI (%PPI,

median and interquartile range) was elicited by three prepulse stimulus levels (65, 75, and 85 dB SPL) at two different ISIs (30 and 100 ms) on four separate days of testing after injection of saline. There was no difference in %PPI for any of the six prepulse conditions in *Cntnap2* WT **(A,** from left to right: Friedman test, 65 dB SPL, 30 ms: p = 0.9651, 65 dB SPL, 100 ms: p = 0.3751; 75 dB SPL, 30 ms: p = 0.6886; 75 dB SPL, 100 ms: p = 0.7661; 85 dB SPL, 30 ms: p = 0.9967; 85 dB SPL, 100 ms: p = 0.9651) or KO rats (**B**, from left to right: Friedman test, 65 dB SPL, 30 ms: p = 0.0642, 65 dB SPL, 100 ms: p = 0.2881; 75 dB SPL, 30 ms: p = 0.8711; 75 dB SPL, 100 ms: p = 0.1039; 85 dB SPL, 30 ms: p = 0.7141; 85 dB SPL, 100 ms: p = 0.9651) within the 2. Treatment Block. This indicated that repeated testing in the course of one treatment block did not systematically alter sensorimotor gating in *Cntnap2* WT or KO rats. Boxplots represent median with interquartile range, and whiskers for maximum and minimum values. Dotted horizontal lines at 0 %PPI represent no PPI of startle.

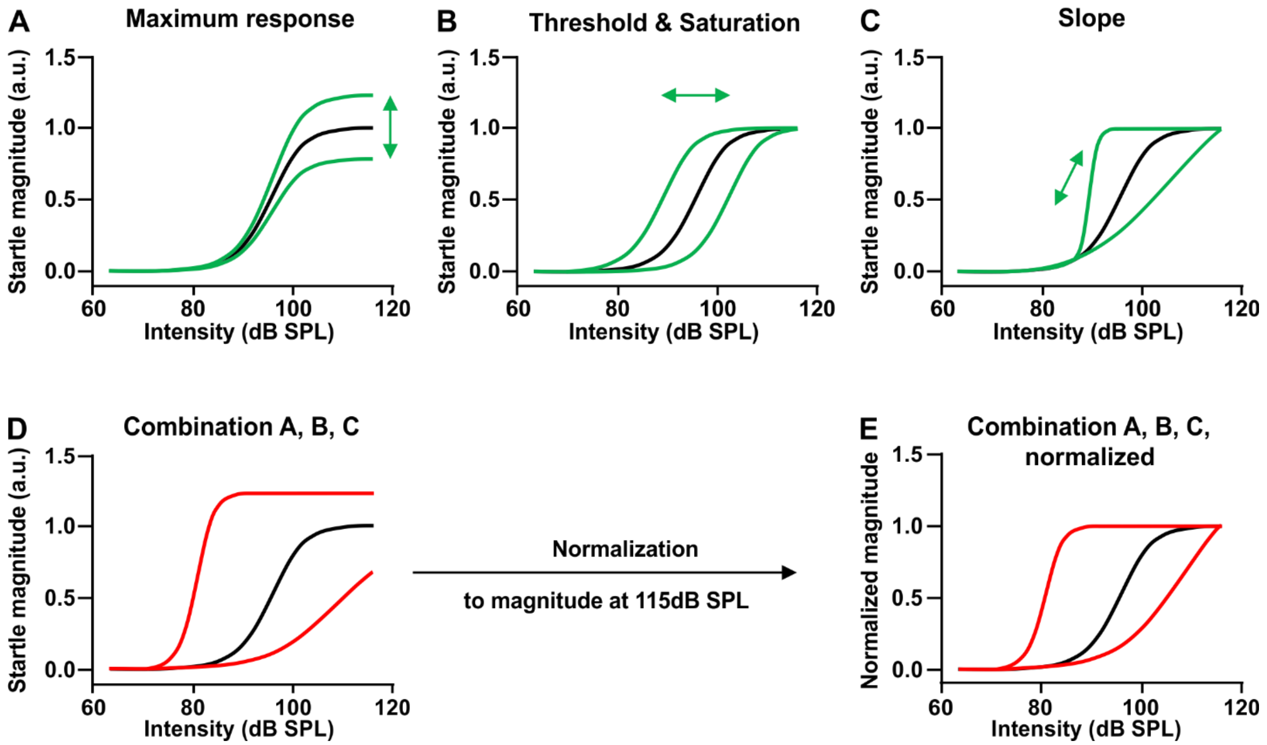

Supplementary Figure 3. Hypothetical plasticity of ASR input-output (I-O) functions. A change in ASR magnitude may be due to **(A)** an increase or decrease in the maximum response obtainable (affecting maximum ASR capacity), **(B)** an increase or decrease in the stimulus intensity required to produce an ASR due to left- or right-shift of the curve (affecting ASR excitability and potency), **(C)** a flattening or steepening of the slope of the dynamic range of the function (affecting ASR efficiency), (**D**) or a combination of these effects (based on data and information from Hince and Martin-Iverson, 2005; Martin-Iverson and Stevenson, 2005). (**E**) Normalization of the ASR I-O function to the individual startle magnitude at the loudest startle pulse allows analysis of threshold and slope without confounding effects of altered maximum response.

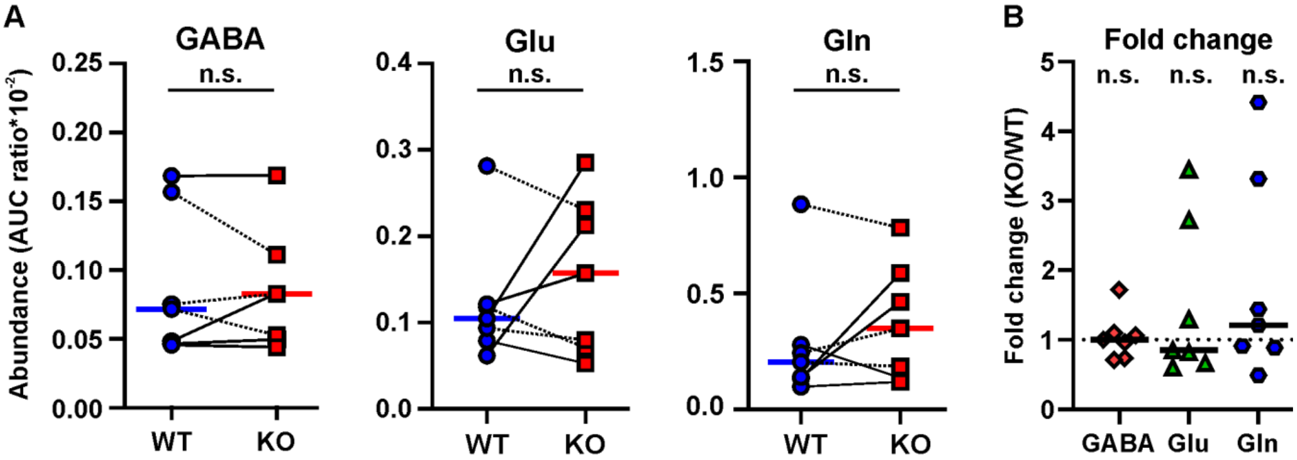

Supplementary Figure 4. Effect of *Cntnap2* knockout on the MALDI MS signals of amino acids in the superior olivary complex (SOC). (A) Pairwise area under the curve (AUC) analysis for *Cntnap2* KO rats ($n = 7$, red squares and horizontal line) compared with WT controls ($n = 7$, blue circles and horizontal line) demonstrated a similar degree of GABA ($[GABA+K]^+$: 142m/z, **Left**, Wilcoxon test $p > 0.9999$), Glutamate ($[Glu+K]^+$: 186 m/z, **Middle**, Wilcoxon test $p = 0.8125$), and Glutamine ($[Gln+ K]^+$: 185m/z, **Right**, Wilcoxon test $p = 0.4688$) signals. Dotted lines denote female, solid lines male WT-KO pairs. **(B)** Comparative analysis showed no fold changes in GABA (one sample Wilcoxon test $p = 0.9375$, median (interquartile range, IQR): 1.00 (0.74-1.10)), Glu (one sample Wilcoxon test $p = 0.8125$, median (IQR): 0.85 (0.67-2.72)), and Gln (one sample Wilcoxon test $p = 0.3750$, median (IQR): 1.21 (0.89-3.31)) levels in the SOC from *Cntnap2* KO rats relative to WT controls. Dotted line at 1 represents no fold change from WT controls. n.s. not significant.

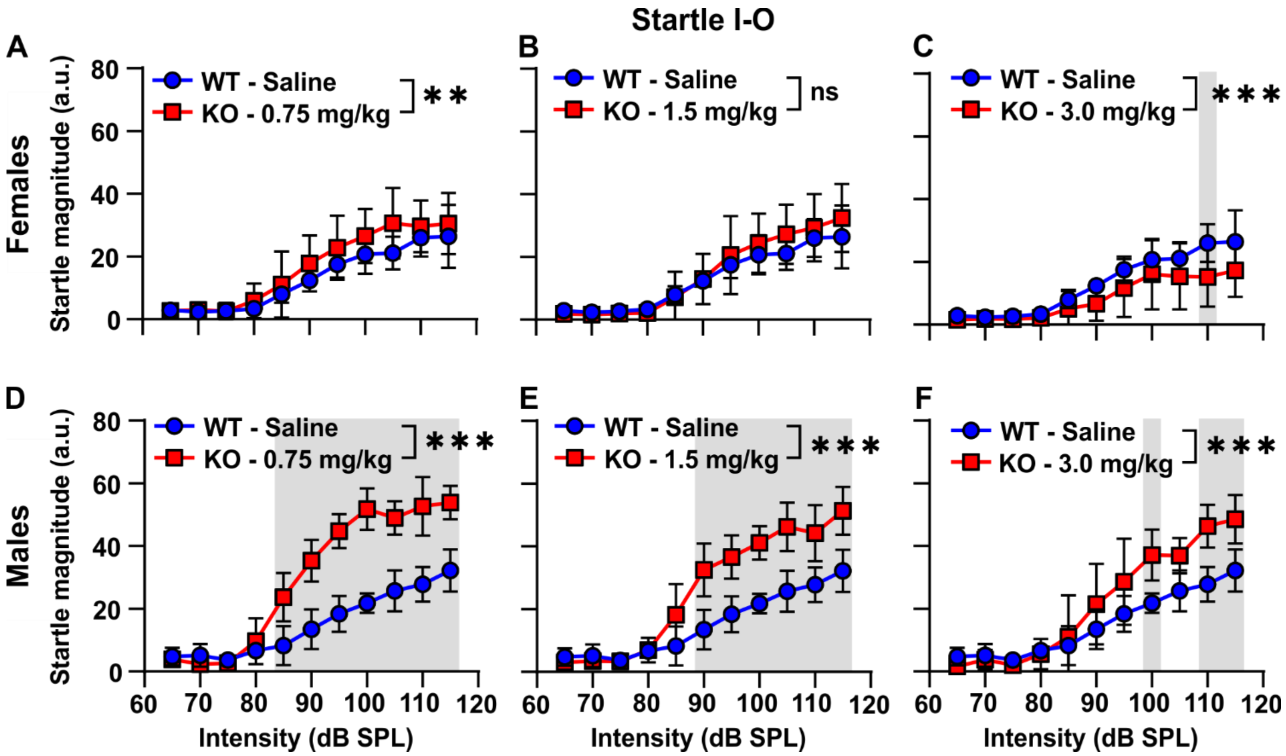

Supplementary Figure 5. R-Baclofen is more effective in *Cntnap2* KO females than males. Mean ± SD startle response magnitudes to increasing startle pulse intensities in *Cntnap2* KO rats (red squares) after injection of R-Baclofen compared with WT rats after saline injection (blue circles). (**A-C**) Compared to female WT rats with saline, ASR magnitudes in female *Cntnap2* KO rats were (**A**) higher with 0.75 mg/kg (WT F: $n = 6$, KO F: $n = 6$, two-way ANOVA, intensity × genotype $p = 0.8163$, $F(10,110) = 0.5935$, intensity $p < 0.0001$, $F(10,110) = 32.17$, genotype $p = 0.0020$, $F(1,110) = 9.996$), (**B**) similar with 1.5 mg/kg (two-way ANOVA, intensity × genotype $p = 0.8372$, $F(10,110) = 0.5676$, intensity $p < 0.0001$, $F(10,110) = 34.01$, genotype $p = 0.1425$, $F(1,110) = 2.181$), (**C**) reduced with 3 mg/kg R-Baclofen (two-way ANOVA, intensity × genotype $p = 0.9894$, $F(10,110) = 0.4570$, intensity $p < 0.0001$, $F(10,110) = 21.85$, genotype $p < 0.0001$, $F(1,110) = 17.92$). (**D-F**) While R-Baclofen dose-dependently reduced the ASR magnitudes in *Cntnap2* KO males (note the reduction of gray shaded areas denoting significantly different magnitudes), none of the three doses brought ASR down to WT controls after saline (WT M: $n = 5$, KO M: $n = 5$, two-way ANOVA, (**D**) intensity × genotype $p < 0.0001$, $F(10,88) = 13.33$, intensity $p < 0.0001$, $F(10,88) = 86.04$, genotype $p < 0.0001$, $F(1,88) = 200.5$; (**E**) intensity × genotype $p < 0.0001$, $F(10,88) = 7.050$, intensity $p < 0.0001$, $F(10,88) = 65.16$, genotype $p < 0.0001$, $F(1,88) = 96.99$). (**F**) Enhanced ASR were particularly robust to higher sound pulse intensities, as seen after injection of 3 mg/kg in *Cntnap2* KO males (two-way ANOVA, intensity × genotype $p = 0.0008$, $F(10,88) = 3.442$, intensity $p < 0.0001$, $F(10,88) = 43.15$, genotype $p < 0.0001$, $F(1,88) = 27.37$). $^{**}p < 0.01$; $^{***}p < 0.001$; n.s. not significant.

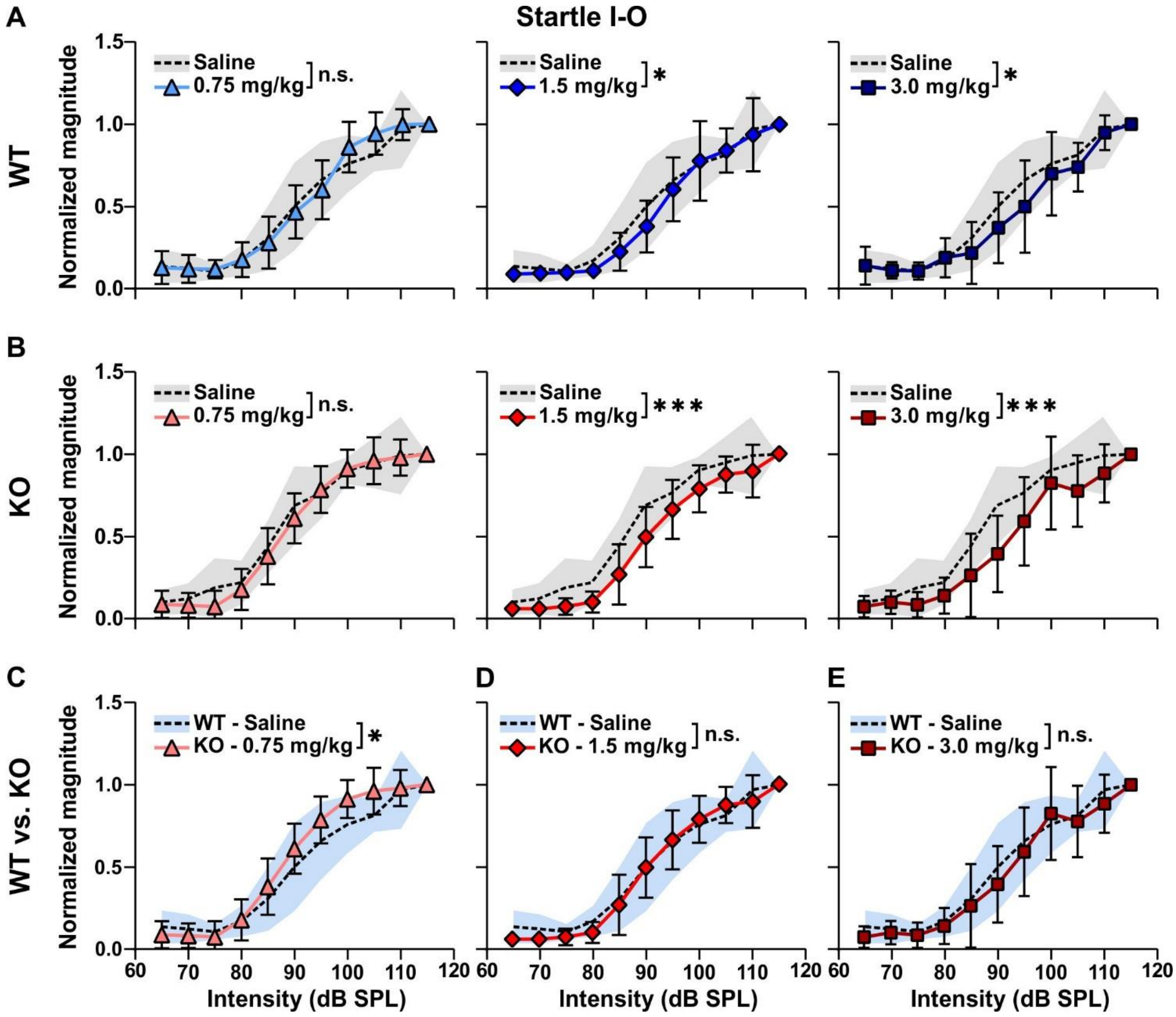

Supplementary Figure 6. Medium and high doses of R-Baclofen decrease ASR magnitudes in *Cntnap2* KO rats to control levels. Mean ± SD startle response magnitudes to increasing startle pulse intensities after injection of saline (dotted line and gray shaded area), 0.75 mg/kg (triangles and error bars), 1.5 mg/kg (diamonds and error bars), 3 mg/kg R-Baclofen (squares and error bars). Magnitudes were normalized for individuals to their magnitude at the loudest sound pulse tested (115 dB SPL) and pooled for females and males. (**A**) In *Cntnap2* WT rats (blue symbols), normalized ASR I-O functions rats were significantly different with 1.5 and 3 mg/kg R-Baclofen compared with saline treatment (*Cntnap2* WT: $n = 11$, two-way RM ANOVA, intensity $p < 0.0001$, $F(10, 110) = 178.9$, treatment $p = 0.0013$, $F(2.735, 300.9) = 5.660$, intensity × treatment $p = 0.5582$, $F(30, 330) = 0.9414$, Dunnett's multiple comparisons test, saline vs. 0.75 mg/kg $p = 0.8280$, saline vs. 1.5 mg/kg $p = 0.0488$, saline vs. 3 mg/kg: $p = 0.0405$). *Post hoc* Sidak's multiple comparisons tests did not show statistically significant differences at a certain startle pulse intensity (dB SPL). (**B**) Normalized ASR I-O functions in *Cntnap2* KO rats were significantly reduced with 1.5 or 3 mg/kg R-Baclofen, in particular at 85 and 90 dB SPL or 90 dB SPL, respectively (*Cntnap2* KO: $n = 11$, two-way RM ANOVA, intensity $p < 0.0001$, $F(10, 110) = 234.5$, treatment $p < 0.0001$, $F(2.393, 263.2) = 17.30$, intensity × treatment $p = 0.3322$, $F(30,330) = 1.100$, Dunnett's multiple comparisons test, saline vs. 0.75 mg/kg $p = 0.1678$, saline vs. 1.5 mg/kg $p < 0.0001$, saline vs. 3 mg/kg:

p < 0.0001; post-hoc two-way RM ANOVA, saline vs. 1.5 mg/kg: intensity *p* < 0.0001, *F*(10, 110) = 133.9, treatment *p* < 0.0001, *F*(1, 110) = 37.24, intensity × treatment *p* = 0.4241, *F*(10, 110) = 1.029, Sidak's multiple comparisons test, 85 dB SPL: *p* = 0.0228, 90 dB SPL: *p* = 0.0057, post-hoc two-way RM ANOVA, saline vs. 3 mg/kg intensity *p* < 0.0001, *F*(10, 110) = 105.4, treatment *p* < 0.0001, *F*(1, 110) = 24.36, intensity × treatment *p* = 0.2363, *F*(10, 110) = 1.306, Sidak's multiple comparisons test, 90 dB SPL: *p* = 0.0017). **(C-E)** Normalized mean ± SD startle response magnitudes to increasing startle pulse intensities in *Cntnap2* KO rats after injection of 0.75 mg/kg (**C**, light red triangles and error bars), 1.5 mg/kg (**D**, red diamonds and error bars), and 3 mg/kg R-Baclofen (**E**, dark red squares and error bars) in comparison with *Cntnap2* WT rats after injection of saline (dotted line and blue shaded area). (**C**) Normalized ASR magnitudes were greater in *Cntnap2* KO rats with 0.75 mg/kg R-Baclofen than in *Cntnap2* WT rats with saline (WT – Saline (*n* = 11) vs. KO – 0.75 mg/kg (*n* = 11), two-way ANOVA, intensity *p* < 0.0001, *F*(10, 220) = 149.9, genotype *p* = 0.0164, *F*(1, 220) = 5.853, intensity × genotype *p* = 0.0973, *F*(10, 220) = 1.638). (**D, E**) Normalized ASR magnitudes are similar in *Cntnap2* WT rats after saline injection and KO rats with 1.5 mg/kg (**D**, WT – Saline (*n* = 11) vs. KO – 1.5 mg/kg (*n* = 11), two-way ANOVA, intensity *p* < 0.0001, *F*(10, 220) = 139.8, genotype *p* = 0.1579, *F*(1, 220) = 2.008, intensity × genotype *p* = 0.8468, *F*(10, 220) = 0.5582) and 3 mg/kg R-Baclofen (**E**, WT – Saline (*n* = 11) vs. KO – 3 mg/kg (*n* = 11), two-way ANOVA, intensity *p* < 0.0001, *F*(10, 220) = 90.54, genotype *p* = 0.0886, *F*(1, 220) = 2.925, intensity × genotype *p* = 0.9536, *F*(10, 220) = 0.3820). **p* < 0.05; ***p* < 0.01; ****p* < 0.001; n.s. not significant.

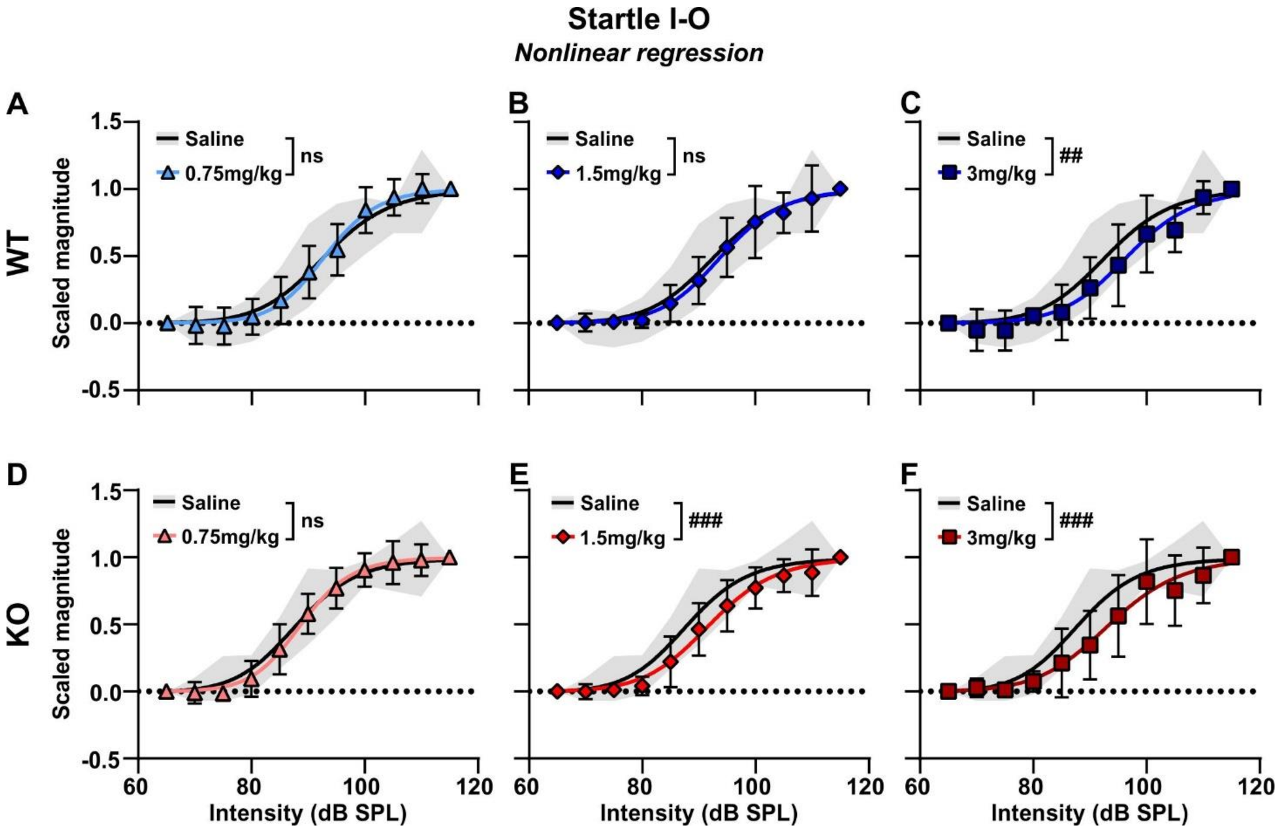

Supplementary Figure 7. Higher doses of R-Baclofen lead to a rightward shift of the startle I-O function. (A-C) Sigmoidal curves (lines) fitted to the startle magnitudes scaled between 0 and 1 in *Cntnap2* WT rats with saline (SD, gray area) and R-Baclofen (mean ± SD, symbols and error bars). The average curve fit was not significantly different with 0.75 mg/kg (**A**, light blue triangles, $p = 0.2896$, $F(2, 238) = 1.246$) and 1.5 mg/kg R-Baclofen (**B**, blue diamonds, $p = 0.3961$, $F(2, 238) = 0.9298$). 3 mg/kg R-Baclofen lead to a significantly different curve fit (**C**, dark blue squares, $p = 0.0029$, $F(2, 238) = 5.988$), resulting from a rightward shift ($p = 0.0007$, $F(1, 238) = 11.92$), whereas the slope remained similar to saline ($p = 0.8063$, $F(1, 238) = 0.06029$). **(D-F)** Sigmoidal curves (lines) fitted to the startle magnitudes scaled between 0 and 1 in *Cntnap2* KO rats with saline (SD, gray area) and R-Baclofen (mean ± SD, symbols and error bars). The average curve fit was not significantly different with 0.75 mg/kg (**D**, light red triangles, $p = 0.1828$, $F(2, 238) = 1.712$). Both 1.5 mg/kg (**E**, red diamonds, $p < 0.0001$, $F(2, 238) = 11.57$) and 3 mg/kg R-Baclofen (**F**, dark red squares and error bars, $p < 0.0001$, $F(2, 238) = 14.06$) lead to significantly different curve fits through a rightward shift with both 1.5 mg/kg (**E**, $p < 0.0001$, $F(1, 238) = 23.06$) and 3 mg/kg R-Baclofen (**F**, $p < 0.0001$, $F(1, 238) = 27.29$). The slopes of the curve fits remained similar to saline with both 1.5 mg/kg (**E**, $p = 0.7693$, $F(1, 238) = 0.08620$) and 3 mg/kg R-Baclofen (**F**, $p = 0.4872$, $F(1, 238) = 0.4843$). Dotted horizontal line denotes the zero line. Comparison of regression lines: ##$p < 0.01$; ###$p < 0.001$; n.s. not significant.

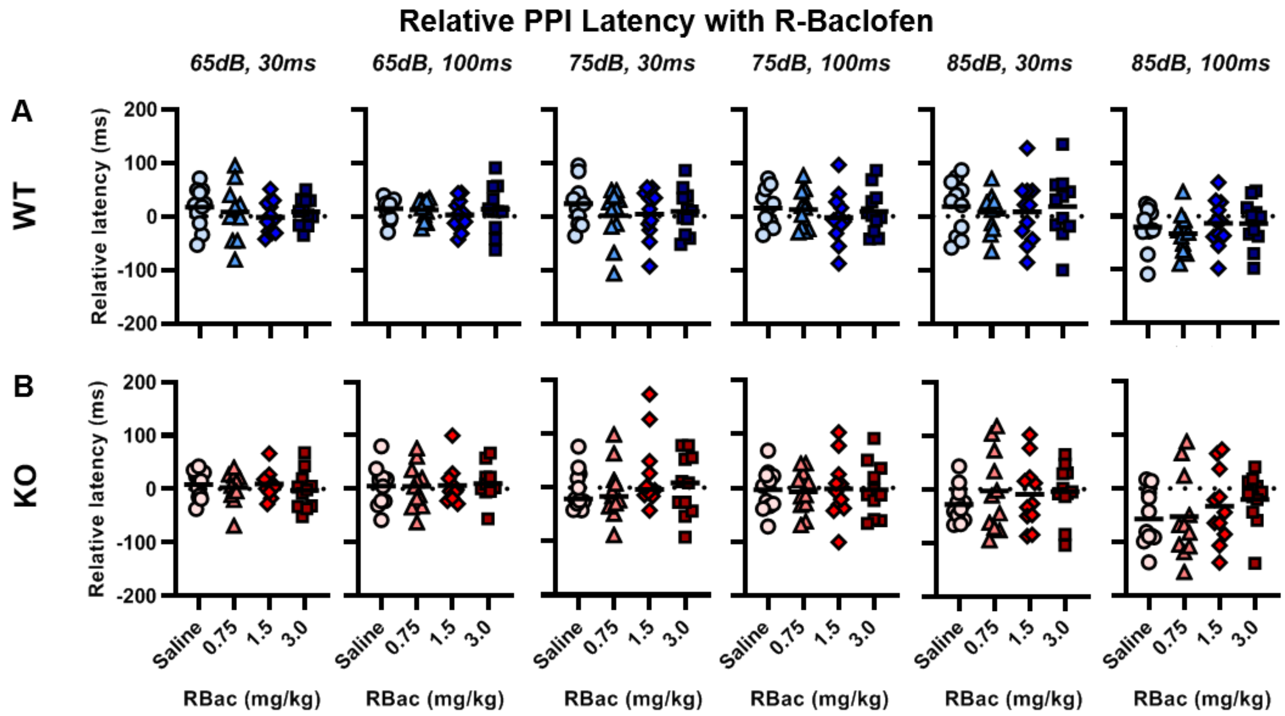

Supplementary Figure 8. Relative latency of PPI in *Cntnap2* WT and KO rats were not significantly altered through R-Baclofen. (A) Scatter plots depicting individual relative latencies in *Cntnap2* WT rats after injection of saline (circles), 0.75 mg/kg (light blue triangles), 1.5 mg/kg (blue diamonds), 3 mg/kg R-Baclofen (dark blue squares). R-Baclofen did not significantly change relative latencies for any of the six prepulse conditions (from left to right: 65 dB SPL, 30 ms ISI, RM one-

way ANOVA, *p* = 0.5693, *F* = 0.6200; 65 dB SPL, 100 ms, RM one-way ANOVA, *p* = 0.7035, *F* = 0.3613; 75 dB SPL, 30 ms, RM one-way ANOVA, *p* = 0.5516, *F* = 0.6916; 75 dB SPL, 100 ms, RM one-way ANOVA, *p* = 0.5880, *F* = 0.5450; 85 dB SPL, 30 ms, RM one-way ANOVA, *p* = 0.7578, *F* = 0.6522; 85 dB SPL, 100 ms, RM one-way ANOVA, *p* = 0.4557, *F* = 0.8732). Black horizontal lines denote mean relative latencies. **(B)** Scatter plots depicting individual relative latencies in *Cntnap2* KO rats after injection of saline (circles), 0.75 mg/kg (light red triangles), 1.5 mg/kg (red diamonds), 3 mg/kg R-Baclofen (dark red squares). R-Baclofen did not significantly increase relative latencies for any of the six prepulse conditions (from left to right: 65 dB SPL, 30 ms, RM one-way ANOVA, *p* = 0.7613, *F* = 0.3609; 65 dB SPL, 100 ms, RM one-way ANOVA, *p* = 0.9652, *F* = 0.05855; 75 dB SPL, 30 ms, Friedman test *p* = 0.5248; 75 dB SPL, 100 ms, RM one-way ANOVA, *p* = 0.9779, *F* = 0.02921; 85 dB SPL, 30 ms, RM one-way ANOVA, *p* = 0.5611, *F* = 0.6374; 85 dB SPL, 100 ms, RM one-way ANOVA, *p* = 0.2701, *F* = 1.388). Black horizontal lines denote median (75 dB SPL, 30 ms) or mean relative latencies. Dotted horizontal lines at 0 Relative latency (ms) represent similar latency to the maximum startle response in trials with versus without a prepulse.

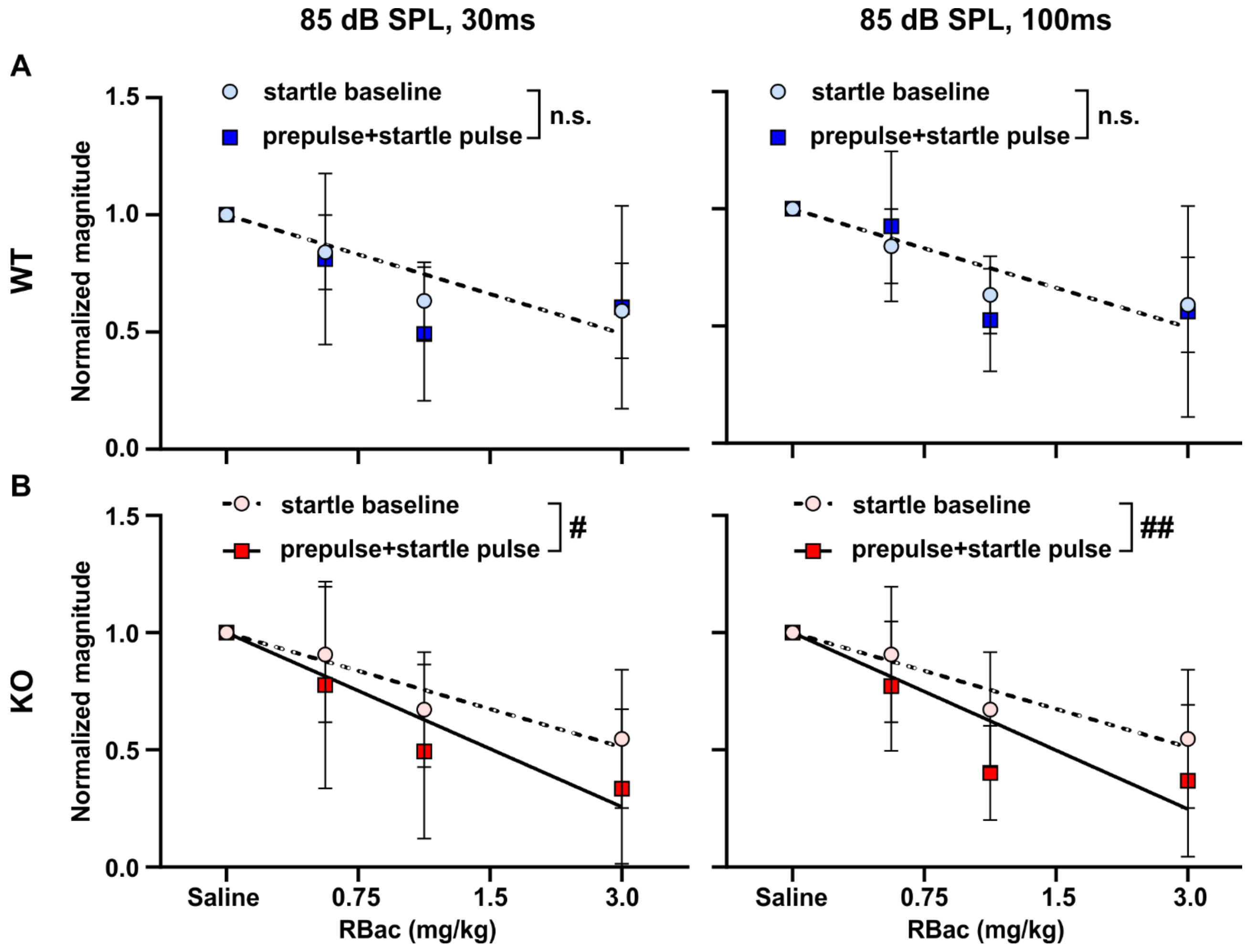

Supplementary Figure 9. Response magnitudes to startle pulses alone (circles) and prepulse+startle pulse trials (squares) across treatment for prepulse conditions with 85dB SPL, 30 ms (left) or 100 ms ISI (right). Magnitudes with R-Baclofen were normalized to that with saline for individual animals and straight-line regression with top constraint to 1. (**A**) In *Cntnap2* WT rats, response magnitudes were uniformly suppressed (*i.e.* shared curve fit) with increasing dose of R-Baclofen to trials with startle pulse alone and trials with prepulse+startle pulse at 30ms ISI (**Left**, straight-line regression, slope $p = 0.6302$, $F(1, 86) = 0.2335$) and at 100ms ISI (**Right**, slope $p = 0.6190$, $F(1, 86) = 0.2490$). (**B**) In *Cntnap2* KO rats the response magnitudes were suppressed more strongly to the prepulse + startle pulse condition than to the startle pulse alone prepulse with 30ms ISI (**Left**, straight-line regression, slope $p = 0.0201$, $F(1, 86) = 5.606$) and with 100ms ISI (**Right**, slope $p = 0.0055$, $F(1, 86) = 8.106$). Comparison of regression lines: #$p < 0.05$; ##$p < 0.01$; n.s. not significant.

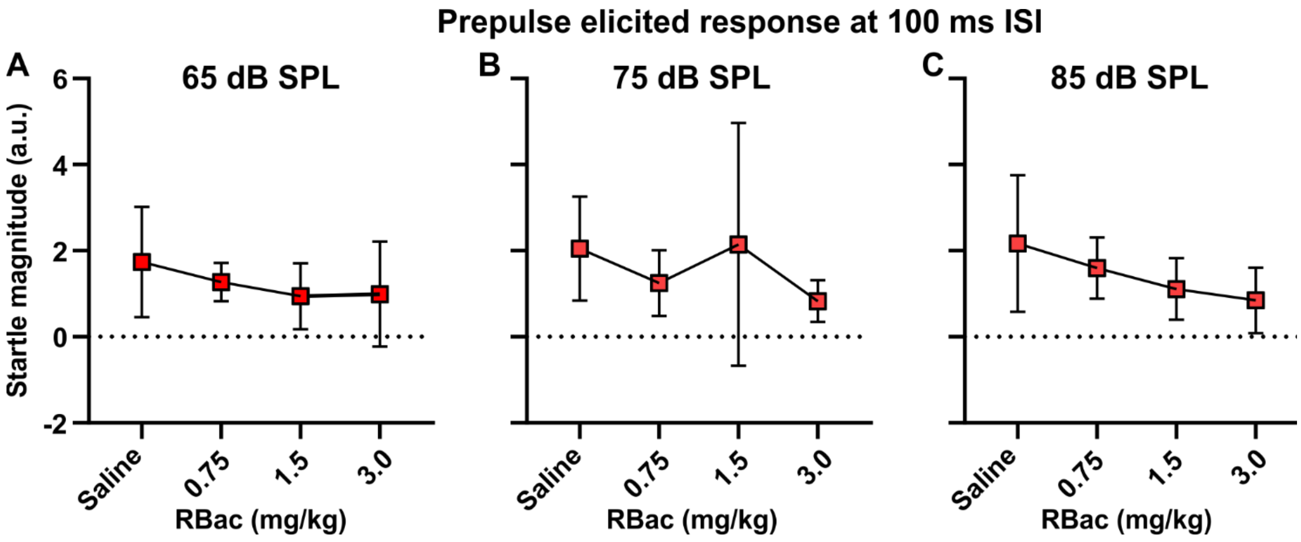

Supplementary Figure 10. Altered %PPI in *Cntnap2* KO rats was not due to increased prepulse elicited response. Motor reaction was not significantly increased in *Cntnap2* KO rats after prepulses with 100 ms ISI (typically occurs within 40 ms after the pulse, Brosda et al. (2011)) at (**A**) 65 dB SPL (Friedman test $p = 0.1381$), (**B**) 75 dB SPL (Friedman test $p = 0.0779$) and (**C**) 85 dB SPL (Friedman test $p = 0.0707$). Dotted line at 0 represents no motor reaction.

2 Supplementary Tables

Supplementary Table 1. Statistical comparison of ASR magnitudes (a.u.) to a series of increasing startle pulse intensities (dB SPL) between R-Baclofen (0.75, 1.5, or 3 mg/kg RBac) and saline within genotype and sex.

		Intensity (dB SPL)										
**RBac vs. saline**		***65***	***70***	***75***	***80***	***85***	***90***	***95***	***100***	***105***	***110***	***115***
***Cntnap2* WT F**	**0.75**	0.9805	0.9986	0.8914	0.9763	0.2064	0.0896	0.1607	0.1042	0.6517	0.0364 *	0.2768
	**1.5**	0.1353	0.8308	0.3295	0.0281 *	0.1477	0.0112 *	0.1058	0.2594	0.2846	0.0024 **	0.0978
	**3.0**	0.0697	0.1929	0.0532	0.0522	0.0239 *	0.0079 **	0.0057 **	0.0098 **	0.0022 **	0.0019 **	0.0176 *
***Cntnap2* WT M**	**0.75**	0.3549	0.3524	0.7939	0.1097	0.6421	0.4081	0.1420	0.8183	0.2479	0.9887	0.3344
	**1.5**	0.1835	0.2219	0.1716	0.0360 *	0.2236	0.0454 *	0.0720	0.0778	0.0879	0.0392 *	0.0372 *
	**3.0**	0.1799	0.2717	0.0895	0.0609	0.0916	0.0102 *	0.0414 *	0.0092 **	0.0177 *	0.0456 *	0.0158 *
***Cntnap2* KO F**	**1.5**	0.2034	0.1710	0.2000	0.0387 *	0.0308 *	0.3730	0.9994	0.7940	0.9193	>0.999	0.6762
	**3.0**	0.1007	0.3315	0.2459	0.1416	0.4332	0.2468	0.2321	0.0728	0.1527	0.0904	0.0787
***Cntnap2* KO F**	**1.5**	0.9927	0.7874	0.0331 *	0.6847	0.2439	0.1956	0.3521	0.2425	0.6638	0.8203	0.9272
	**3.0**	0.6446	0.9627	0.0134 *	0.2267	0.0794	0.1017	0.0443 *	0.3616	0.1871	0.7096	0.5122

Dunnett's multiple comparisons tests, p values, *$p < 0.05$, **$p < 0.01$

Supplementary Table 2. ASR I-O mean threshold and median saturation (dB SPL) in *Cntnap2* WT and KO rats calculated from sigmoidal regression after saline or RBac treatment with 0.75, 1.5, or 3 mg/kg administration.

	RBac (mg/kg)	***Saline***	***0.75***	***1.5***	***3***
Threshold (dB SPL)	***Cntnap2* WT**	87.9	88.1	89.4	92.2
	***Cntnap2* KO**	82.8	84.3	86.5	88.8
**∆*Threshold (dB)***	**KO vs. WT Saline**	-5.1	-3.5	-1.4	0.9
Saturation (dB SPL)	***Cntnap2* WT**	109.3	100.7	104.3	111.3
	***Cntnap2* KO**	100.8	97.2	109.3	104.0
**∆*Saturation (dB)***	**KO vs. WT Saline**	-8.5	-12.1	0.01	-5.3

∆Threshold (dB) and ∆Saturation (dB) were calculated by subtracting the ASR threshold or saturation in *Cntnap2* WT rats after saline treatment from.the respective acoustic startle pulse intensity in *Cntnap2* KO rats after Saline or RBac treatment with 0.75, 1.5, or 3 mg/kg.

Supplementary Table 3. Comparison of sigmoidal regression fits of ASR IO functions with magnitude scaled between 0 and 1 within *Cntnap2* WT or KO rats.

	Rbac (mg/kg)	***Saline***	***0.75***	***1.5***	***3***
	**Bottom**	=0	=0	=0	=0
	**Top**	=1	=1	=1	=1
***Cntnap2* WT**	**ES50**	92.92	92.87	94.10	96.67
	**HillSlope**	15.92	19.96	17.41	16.52
	**Sy.x**	0.1978	0.1400	15.06	0.1809
	**Different curve fits?**	N/A	0.2896<br>n.s.	0.3961<br>n.s.	0.0029<br>★★
**WT – Saline vs. WT – RBac**	**Different slopes?**	N/A	0.1164<br>n.s.	0.5407<br>n.s.	0.8063<br>n.s.
	**Different ES50?**	N/A	0.9570<br>n.s.	0.2281<br>n.s.	0.0007<br>★★★
	**Bottom**	=0	=0	=0	=0
	**Top**	=1	=1	=1	=1
***Cntnap2* KO**	**ES50**	87.96	88.82	91.97	93.47
	**HillSlope**	16.31	19.85	15.70	14.60
	**Sy.x**	0.1688	0.1184	0.1304	0.1999
	**Different curve fits?**	N/A	0.1828	<0.0001<br>★★★	<0.0001<br>★★★
**KO – Saline vs. KO – RBac**	**Different slopes?**	N/A	0.1407	0.7693	0.4872
	**Different SC50?**	N/A	0.2586	<0.0001<br>★★★	<0.0001<br>★★★

Bottom plateau constraint to 0, Top plateau constraint to 1, ES50: acoustic pulse intensity (dB SPL) that gives a startle magnitude halfway between Bottom and Top, HillSlope: steepness of the curve, Sy.x: standard error of regression, KO vs. WT – Saline: curve fit comparison between *Cntnap2* KO rats treated with saline, 0.75, 1.5, or 3 mg/kg R-Baclofen and WT rats with saline. *p* values, **$p < 0.01$, ***$p < 0.001$, n.s. not significant

3 Supplementary References

Brosda, J., Hayn, L., Klein, C., Koch, M., Meyer, C., Schallhorn, R., Wegener, N., 2011. Pharmacological and parametrical investigation of prepulse inhibition of startle and prepulse elicited reactions in Wistar rats. Pharmacol Biochem Behav 99, 22-28.

Hince, D.A., Martin-Iverson, M.T., 2005. Differences in prepulse inhibition (PPI) between Wistar and Sprague-Dawley rats clarified by a new method of PPI standardization. Behav Neurosci 119, 66-77.

Martin-Iverson, M.T., Stevenson, K.N., 2005. Apomorphine effects on emotional modulation of the startle reflex in rats. Psychopharmacol (Berl) 181, 60-70.
